# Supplementary material for: Early postnatal expression mitigates immune responses to Cas9 in the murine central nervous system
Source: Mol Ther Methods Clin Dev. 2025 Jul 17;33(3):101536. doi: 10.1016/j.omtm.2025.101536 (PMC12347139; doi:10.1016/j.omtm.2025.101536)
Supplement: Document S1. Figures S1–S5 and Table S1 [file mmc1.pdf]

OMTM, Volume *33*

Supplemental information

Early postnatal expression mitigates immune responses to Cas9 in the murine central nervous system

Robert Duba-Kiss and David R. Hampson

Table S1. List of primers used in RT-qPCR gene expression experiments.

Target Gene	Forward Primer	Reverse Primer
Ppia	CGTCTCCTTCGAGCTGTTTG	CCACATGCTTGCCATCCAG
Pgk1	CTAGAGCTCCTGGAAGGTAAAG	GAGAGCTCAGCCTTTACAGC
S. aureus Cas9	GTACGGCGACGAGAAGAATC	ACTTGTACACGCCATTGTCC
Tnfα	CCCAAAGGGATGAGAAGTTCC	AGATAGCAAATCGGCTGACG
Il1β	TGCCACCTTTTGACAGTGATG	ATGTGCTGCTGCGAGATTTG
Ccl2	CCTGCTGCTACTCATTCACCA	CTTGAGCTTGGTGACAAAAACTACA
Ifnγ	ACTGGCAAAAGGATGGTGAC	GTGGGTTGTTGACCTCAAAC
Il10	GCTCTTACTGACTGGCATGAG	CGCAGCTCTAGGAGCATGTG
Tgfβ	TGGAAATCAACGGGATCAGC	TAGTTGGTATCCAGGGCTCTC

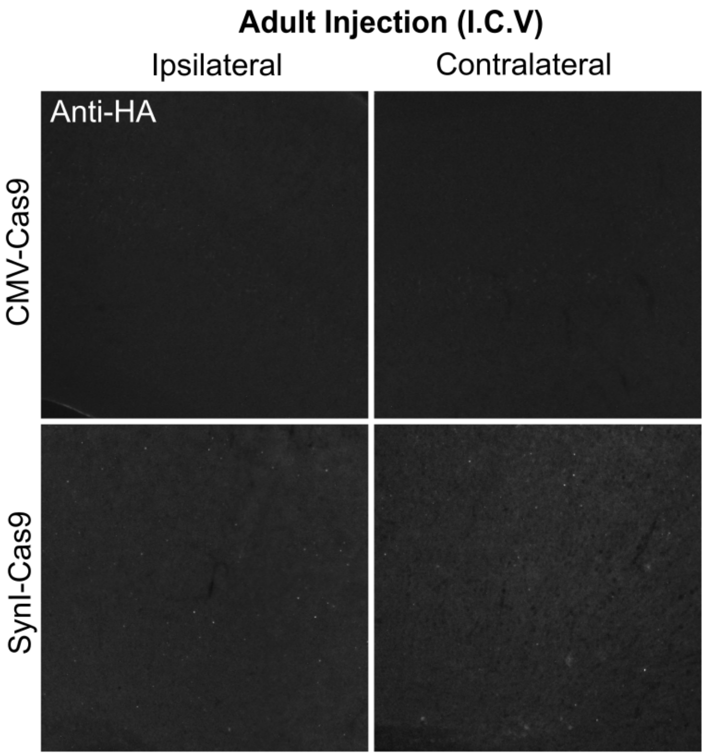

Figure S1. Representative low-magnification images showing lack of immunostaining using anti-HA in the somatosensory cortices of mice injected with Cas9-HA-encoding AAVs by i.c.v. injection as adults. Mice injected as neonates with AAV-SynI-Cas9 or AAV-CMV-Cas9 were used as positive controls for Cas9-HA expression analyses of adult-injected mice (not shown).

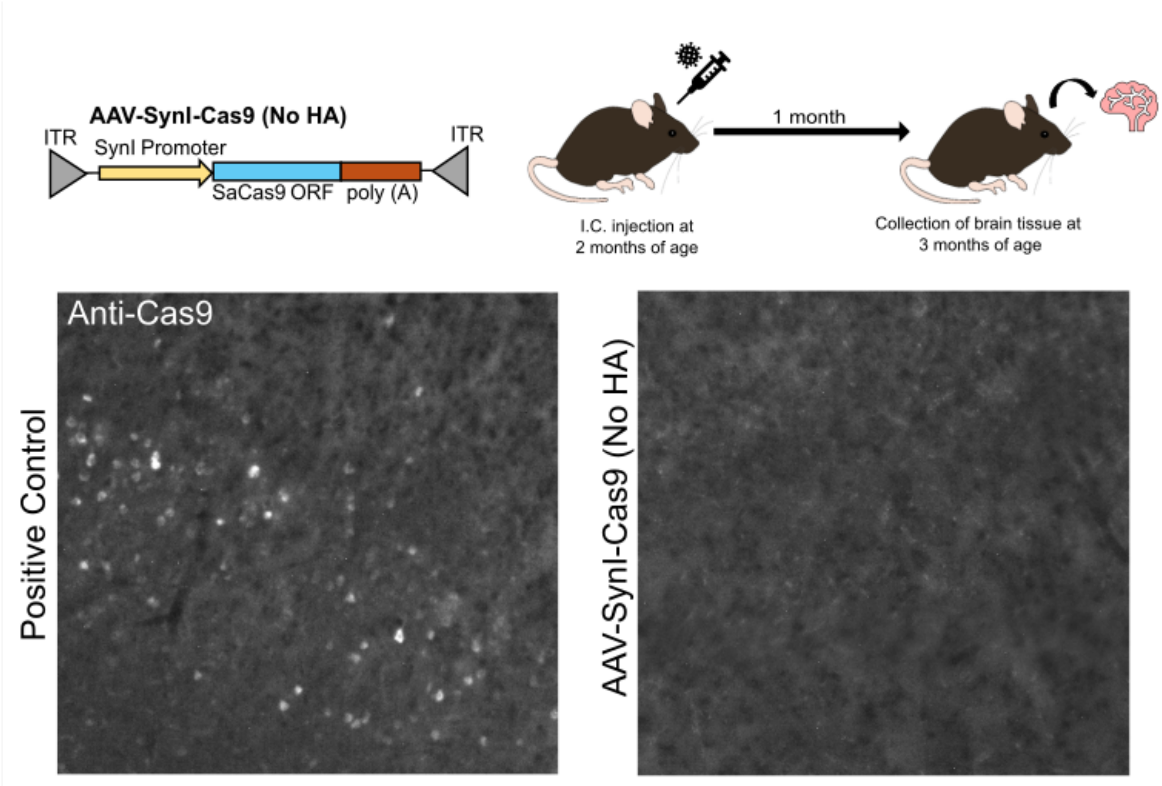

Figure S2. Representative low-magnification image of the somatosensory cortex of a mouse injected with AAV-SynI-Cas9 lacking an HA tag. Adult mice were given an intra-cortical injection with AAV-SynI-Cas9 (no HA) and the brains were collected 28 days post-injection (n = 6). Brain sections were probed with an anti-Cas9 antibody. The AAV dose used was 9.12 x 10^{10} GCs. The positive controls were brain sections from mice injected with AAV-SynI-Cas9 (with an HA tag) as neonates and were also probed with the anti-Cas9 antibody. HA: hemagglutinin; I.C.: intracortical; ITR: inverted terminal repeats; ORF: open reading frame.

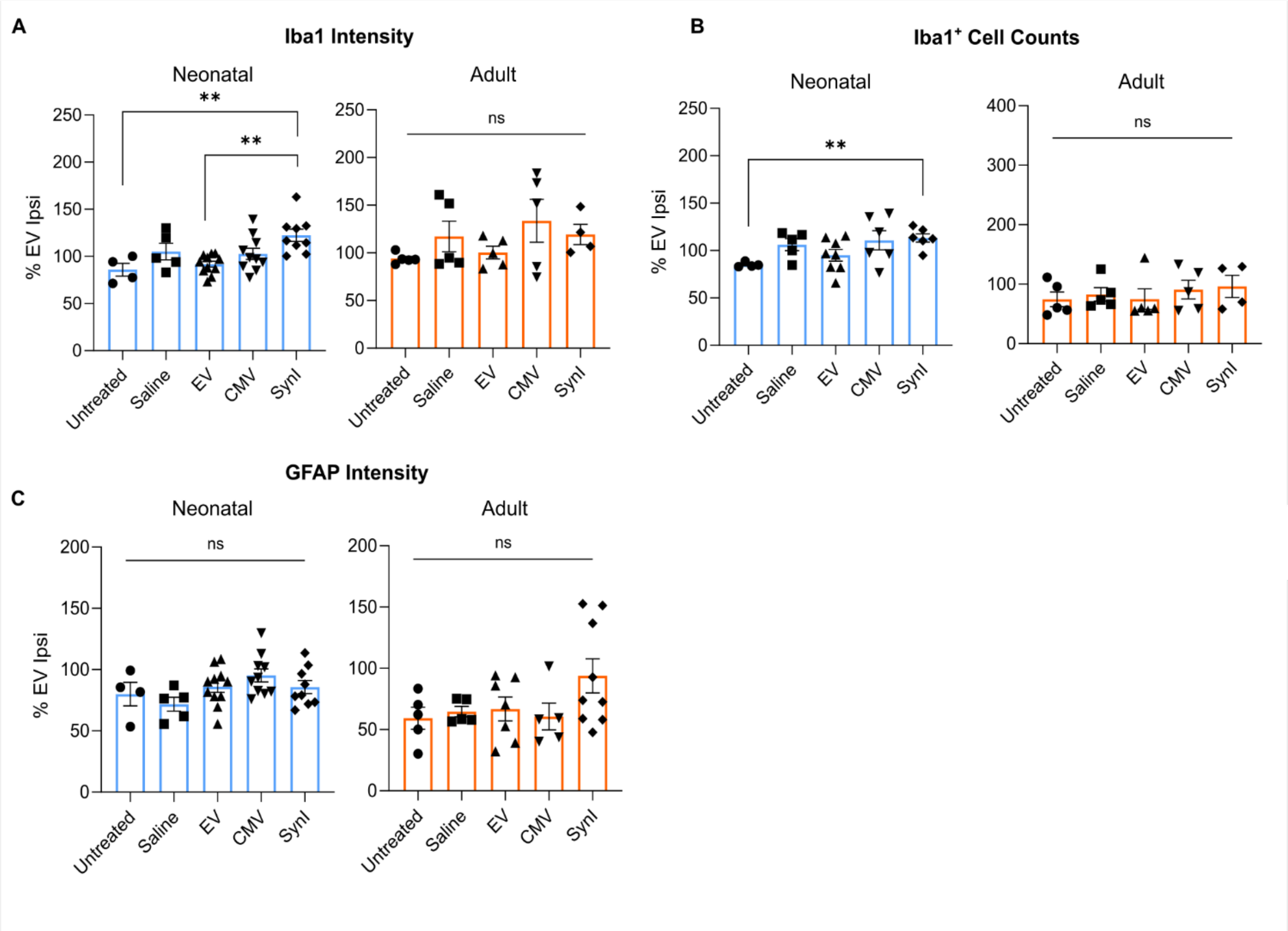

Figure S3. Quantitative analyses of Iba1 fluorescence intensity (A) and Iba1 cell counts (B) in the contralateral somatosensory cortices of mice administered AAV as neonates or adults. (C) Quantitative analyses of GFAP fluorescence intensity in the contralateral somatosensory cortices of mice administered AAV as neonates or adults. ns: not significant; ** p<0.01, Tukey *post hoc* or Dunnett's multiple comparison test.

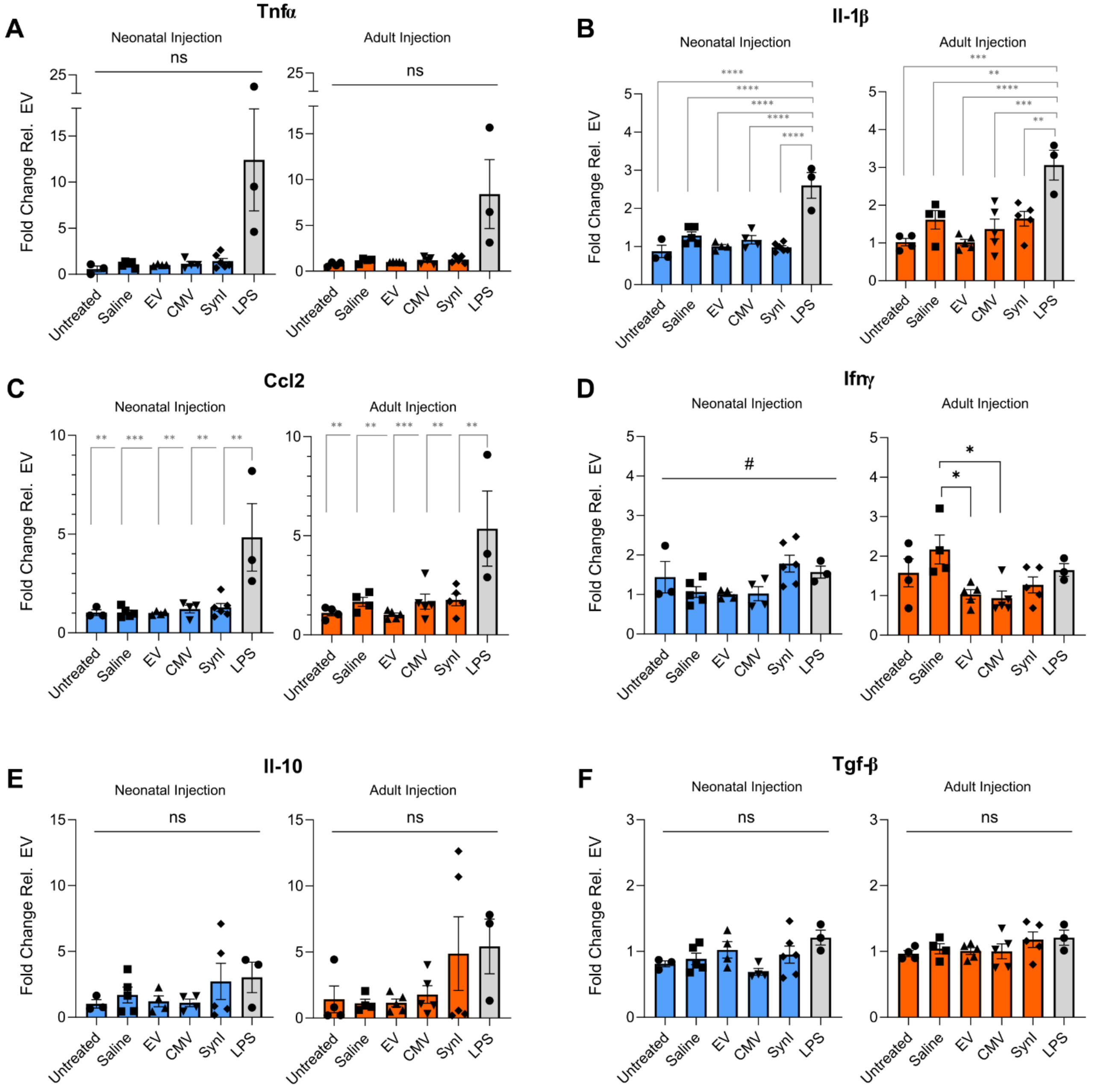

Figure S4. Quantification of cytokine expression in cerebral cortices one month following AAV treatment. Proinflammatory cytokines *Tnfα* (A), *Il-1β* (B), *Ccl2* (C), and *Ifnγ* (D), and anti-inflammatory cytokines *Tgf-β* (E) and *Il-10* (F). Cortical extracts from adult mice administered 2 mg/kg of LPS by intraperitoneal injection were used as a control (grey). ns: not significant; #: $p < 0.05$, one-way ANOVA; *: $p < 0.05$, **: $p < 0.01$, ***: $p < 0.001$, ****: $p < 0.0001$, Tukey's *post hoc* test.

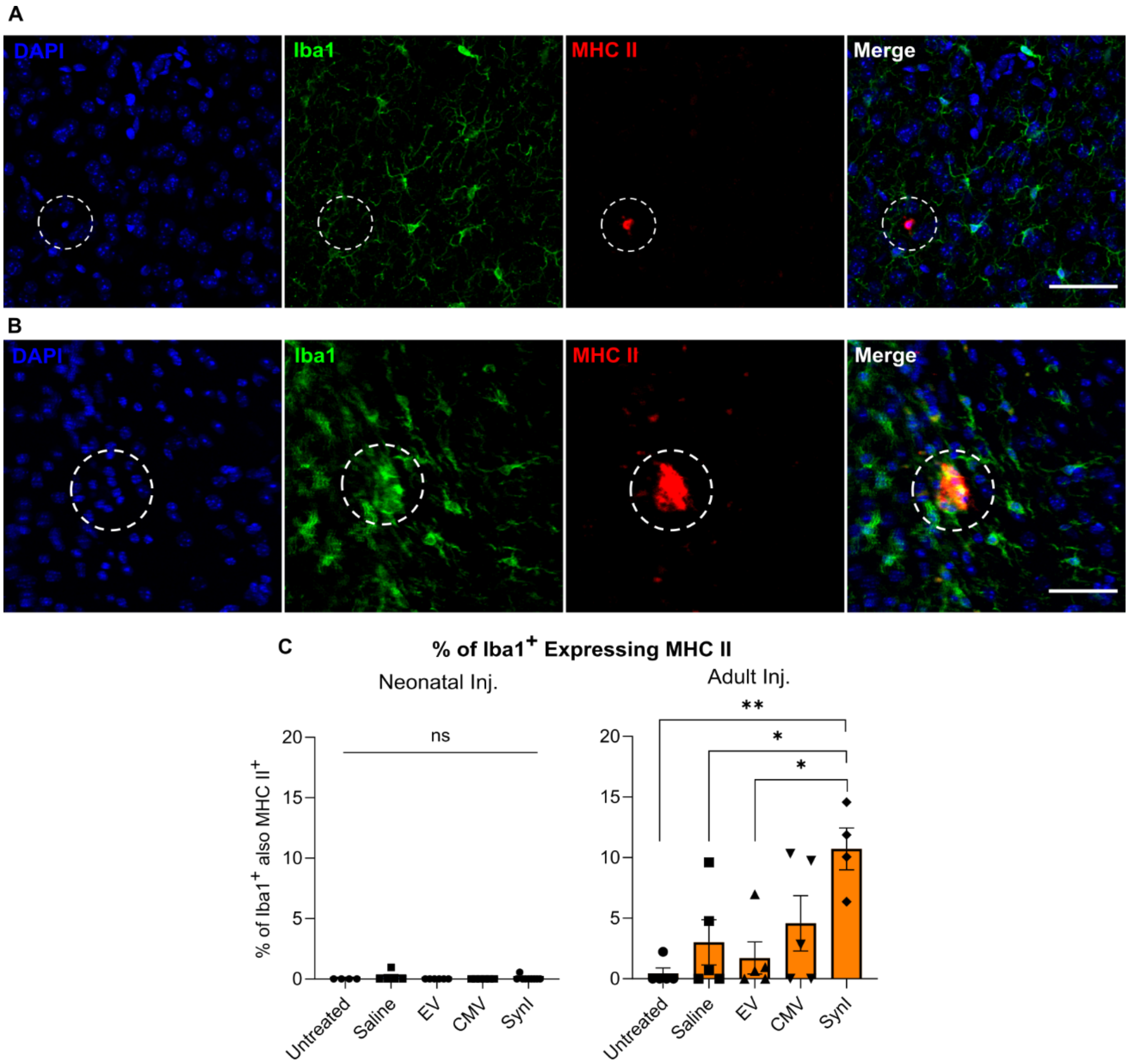

Figure S5. Representative images of double immunolabeling of Iba1 (green) and MHC II (red) in the ipsilateral somatosensory cortices of mice administered AAV-SynI-Cas9 as (A) neonates and (B) adults. Scale bars = 50 µm. (C) Quantitative results of co-labeling of $Iba1^+$ microglia and MHC II. ns: not significant; * $p < 0.05$, ** $p < 0.01$, Tukey's *post hoc* test.
