# Supplementary material for: Early postnatal expression mitigates immune responses to Cas9 in the murine central nervous system
Source: Mol Ther Methods Clin Dev. 2025 Jul 17;33(3):101536. doi: 10.1016/j.omtm.2025.101536 (PMC12347139; doi:10.1016/j.omtm.2025.101536)
Supplement: Document S2. Article plus supplemental information [file mmc2.pdf]

Molecular Therapy
Methods & Clinical Development
Original Article

Early postnatal expression mitigates immune responses to Cas9 in the murine central nervous system

Robert Duba-Kiss[1] and David R. Hampson[1,2]

[1]Department of Pharmacology and Toxicology, University of Toronto, Toronto, ON, Canada; [2]Department of Pharmaceutical Sciences, University of Toronto, Toronto, ON, Canada

A barrier in the development of adeno-associated virus (AAV) gene therapy is the immunogenicity of the AAV particles, and in some cases, the expressed transgene. The immunogenic risk is heightened when exogenous proteins, such as prokaryotic Cas9 nucleases, are used in gene editing. We documented the immune responses generated after CNS injections of AAVs encoding *Staphylococcus aureus* Cas9 in neonatal vs. adult mice. Injection of AAV-Cas9s containing either a neuron-specific synapsin promoter, or a cytomegalovirus promoter, into neonatal mice resulted in robust expression of Cas9 and activation of microglia. Expression of Cas9 was maintained 3 months post-injection while the microglial response dissipated by this time. In contrast, infusion of AAV-Cas9s into the adult brain resulted in the absence of detectable Cas9 protein and reduced neuronal density. Activation of microglia and astrocytes, T cell infiltration into the CNS, and circulating antibodies were also observed. Surprisingly, these effects were not seen in adult mice administered an AAV encoding EGFP. The lack of detectable Cas9 protein in the adult injected mice indicates that Cas9-expressing neurons were eliminated via a cytotoxic immune response. Our findings suggest that very early postnatal administration may enhance safety and efficacy in treatments for pediatric disorders employing CRISPR-Cas9 gene editing.

INTRODUCTION

Adeno-associated virus (AAV)-mediated gene therapies are being explored as candidate treatments for a wide range of neurological disorders. Although the unique advantages of this approach offer promise for diseases for which conventional pharmacological interventions have not been effective, critical roadblocks must nevertheless be overcome before such treatments become prevalent in clinical use. These challenges include the immunogenicity of the viral capsid proteins, the viral genome, and transgene product; inflammatory responses elicited by any of these components may result in the elimination of cells transduced by the viral vector, negating any therapeutic benefit of the treatment. In some cases, severe immune responses to AAV therapy have led to life-threatening or fatal adverse inflammatory events.[1,2]

Some AAV-mediated therapies in development for central nervous system (CNS) disorders make use of non-endogenous therapeutic proteins. These may include proteins that are human in origin, but which contain epitopes that the patient does not produce due to nonsense or frameshift mutations,[3] or proteins that are derived from different species entirely. A salient example of the latter is the prokaryotic CRISPR-associated protein 9 (Cas9) nuclease used in gene editing and gene knockout.[4–6] Several studies have characterized the immunogenicity of Cas9[7–10]; importantly, the presence of pre-existing immunological memory to Cas9 (i.e., Cas9-reactive antibodies and T cells) has been reported in humans and other mammals,[11–14] which can potentiate Cas9-directed inflammatory responses.[15] Although the CNS has previously been considered to be an "immune-privileged" compartment in which the induction of immune responses is tightly regulated, more recent findings have shown that the expression of foreign proteins in the brain by AAV gene transfer elicits inflammatory and cytotoxic responses.[8,16–20] Therefore, there is a need to develop treatment strategies to mitigate the immunogenic risks of using non-self proteins before they may be safely and widely adopted for clinical use.

Effective treatments for some neurodevelopmental disorders may require drug administration, including biologically based agents such as viral vectors, to newborns or very young infants. During fetal and early postnatal development, the immune system is uniquely programmed to develop tolerance to non-self antigens, as it must become accustomed to maternal alloantigens, commensal microbes, and harmless antigens from the environment. This neonatal "window of opportunity" could theoretically be exploited to induce immune tolerance to foreign proteins by administering AAVs shortly following birth. Several reports have shown that exposure to AAVs encoding non-self proteins to neonatal mice or rats (but not adults) can circumvent triggering humoral and cellular adaptive immune responses.[21–23] However, the full consequences of neuroinflammatory

Received 22 May 2025; accepted 12 July 2025;
https://doi.org/10.1016/j.omtm.2025.101536.

Correspondence: David R. Hampson, Department of Pharmacology and Toxicology, University of Toronto, Toronto, ON, Canada.
E-mail: d.hampson@utoronto.ca

Published by Elsevier Inc. on behalf of The American Society of Gene and Cell Therapy.
This is an open access article under the CC BY license (http://creativecommons.org/licenses/by/4.0/).

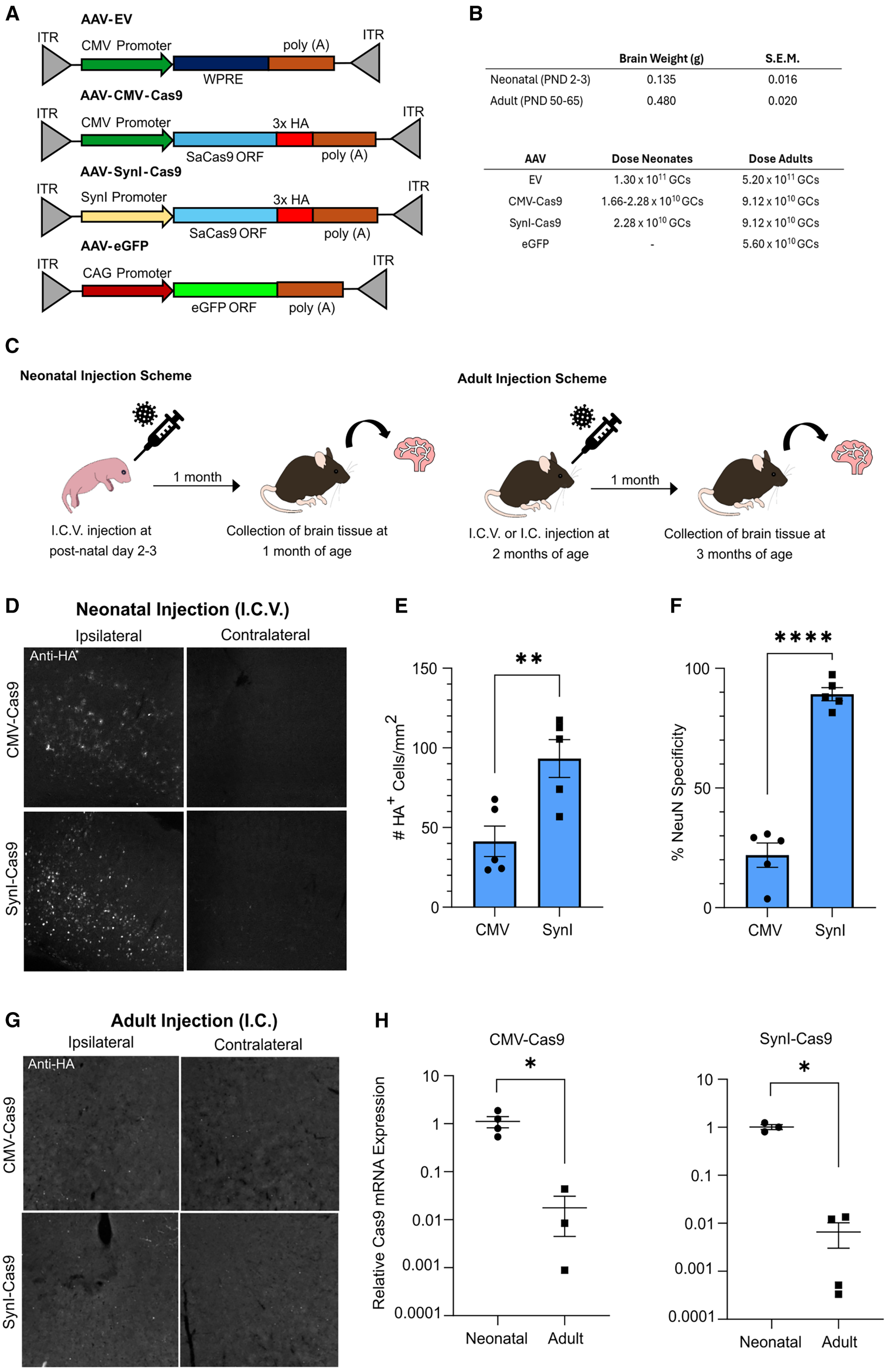

	Brain Weight (g)	S.E.M.
Neonatal (PND 2-3)	0.135	0.016
Adult (PND 50-65)	0.480	0.020

AAV	Dose Neonates	Dose Adults
EV	1.30 x 10^{11} GCs	5.20 x 10^{11} GCs
CMV-Cas9	1.66-2.28 x 10^{10} GCs	9.12 x 10^{10} GCs
SynI-Cas9	2.28 x 10^{10} GCs	9.12 x 10^{10} GCs
eGFP	-	5.60 x 10^{10} GCs

(legend on next page)

responses after direct injection of AAV-Cas9s into the early postnatal brain vs. the adult brain remain unknown.

In this study, we administered AAVs encoding *Staphylococcus aureus* Cas9 to the neonatal and adult mouse CNS and examined multiple markers of neuroinflammation and neurotoxicity. Infusions of AAV-Cas9 into the adult mouse brain resulted in the strong upregulation of immune markers, loss of neurons, and the absence of Cas9-expressing cells, while CNS injections of the same AAVs to neonatal mouse pups led to robust Cas9 expression and a relatively subdued upregulation of immune markers. We conclude that exposure during early postnatal development effectively reduces the immunogenicity of Cas9 within the CNS. These findings are particularly relevant to AAV gene therapies for neurodevelopmental disorders, where treatment shortly following birth may not only promote the normalization of brain development, but may also mitigate immune activation and neurotoxicity, and promote therapeutic efficacy.

RESULTS

Cas9-HA expression and neurotoxicity

Two AAV vectors (serotype 9) encoding Cas9 were used in this study; one incorporated a neuron-selective human synapsin I promoter (AAV-SynI-Cas9), while the other contained a cytomegalovirus promoter (AAV-CMV-Cas9; Figure 1A). Both constructs encoded *S. aureus* Cas9 protein with a C-terminal hemagglutinin (HA) tag, followed by a bovine growth hormone 3′ untranslated region sequence. The doses of these two vectors were similar (Figure 1B); however, because the murine brain is roughly four times larger at 2 months of age compared with postnatal day 2 (PND 2),[24] the doses administered to adult mice were four times higher than to neonatal mice (Figure 1B). Except in the experiments assessing longer-term (3 months post-injection) Cas9 expression and cellular marker levels, brain tissue was collected 1 month after injection and analyzed by immunofluorescence (IF) staining using anti-HA (Figure 1C).

Strong expression of Cas9-HA was detected 1 month post-injection in the ipsilateral somatosensory and motor cortices of mice administered AAV-SynI-Cas9 or AAV-CMV-Cas9 following intracerebroventricular (i.c.v.) injection at PND 2 or 3 (Figure 1D, left panels). No expression was detected in the contralateral cortices (Figure 1D; right panels). More Cas9-HA$^+$ cells were detected following injection of the AAV-SynI-Cas9 construct than the AAV-CMV-Cas9 (Figure 1E). Double-label IF staining with anti-HA and anti-neuronal nuclear antigen (NeuN) revealed that, as anticipated, AAV-SynI-Cas9 primarily restricted Cas9-HA expression to NeuN$^+$ neurons, whereas AAV-CMV-Cas9 elicited expression mainly in non-neuronal cells, likely glia such as astrocytes (Figure 1F). In contrast, neither i.c.v. (Figure S1) nor direct intracortical (i.c.) administration of AAV-SynI-Cas9 or AAV-CMV-Cas9 to the adult brain resulted in Cas9-HA expression in the cerebral cortex (Figure 1G). However, Cas9-HA mRNA was detected by RT-qPCR in the cerebral cortex following adult i.c. injection of both AAV-SynI-Cas9 and AAV-CMV-Cas9, although in both cases the transcript abundance was very low relative to samples from mice injected neonatally (Figure 1H).

Based on these findings, we theorized that the lack of detectable Cas9-HA protein expression in the adult injected cohort might have been the result of an inflammatory response mobilized against the transgenic Cas9 protein antigen, which had eliminated Cas9-expressing cells prior to sample collection. Because we were unable to detect Cas9-HA expression after adult AAV injections by IF staining, it would have been difficult to conclusively identify cortical areas that the virus had transduced following i.c.v. injection. Therefore, in subsequent analyses of immune markers, we elected to administer AAVs to adult mice by i.c. injection and conduct all analyses near the injection position; doing so allowed us to consistently analyze a region of the cerebral cortex that encompassed the injected virus.

The HA tag, which was incorporated into the Cas9-HA protein, is derived from the hemagglutinin protein from the human influenza virus and may exhibit immunogenic properties.[25] Although this tag only made up about 2% of the total polypeptide, we nevertheless asked whether its use may have augmented the immune response to Cas9 and led to the observed lack of expression following AAV administration to adult mice. To test this, we custom synthesized an AAV that contained the SynI promoter and SaCas9 coding sequence but did not contain the HA tag. We injected this construct into the adult mouse cerebral cortex and assessed whether Cas9$^+$ cells were detectable using an anti-Cas9 antibody. As observed with the Cas9-HA vector, no Cas9$^+$ cells were observed 4 weeks after injection (Figure S2). We conclude that the immunological elimination of neurons after injecting Cas9 in the adult mouse brain holds true irrespective of whether or not an HA tag is present.

Figure 1. Experimental design and Cas9-HA expression in the cerebral cortex

(A) AAV vector constructs. (B) Weights of neonatal and adult mouse brains and doses of AAVs administered. (C) Injection and tissue collection timelines. (D) Representative examples of Cas9-HA protein expression in the ipsilateral and contralateral somatosensory cerebral cortices of mice administered Cas9-encoding AAVs as neonates by unilateral i.c.v. injection. (E) Counts of Cas9-HA$^+$ cells in the ipsilateral somatosensory cortices of mice injected as neonates. $^{**}p < 0.01$, unpaired t test. (F) Specificity of Cas9-HA expression in mice injected as neonates to NeuN$^+$ neurons in the somatosensory cortex. Specificity is calculated as the number of cells with overlapping NeuN and HA signal, divided by the total number of cells expressing HA. $^{****}p < 0.0001$, unpaired t test. (G) Representative examples of Cas9-HA protein expression in the ipsilateral and contralateral somatosensory cerebral cortices of mice administered Cas9-encoding AAVs as adults by unilateral i.c. injection. (H) Quantification of Cas9 mRNA in cerebral cortex 1 month following i.c.v. AAV injection of neonatal and i.c. AAV injection of adult mice. $^{*}p < 0.05$, unpaired t test with Welch's correction. CMV, cytomegalovirus; eGFP, enhanced green fluorescent protein; EV, empty vector; GC, genome copies; HA, hemagglutinin; I.C.V., intracerebroventricular; I.C., intracortical; ITR, inverted terminal repeats; ORF, open reading frame; SaCas9, *Staphylococcus aureus* CRISPR-associated protein 9; S.E.M., standard error of the mean; SynI, human synapsin I; WPRE, woodchuck hepatitis virus posttranscriptional regulatory element.

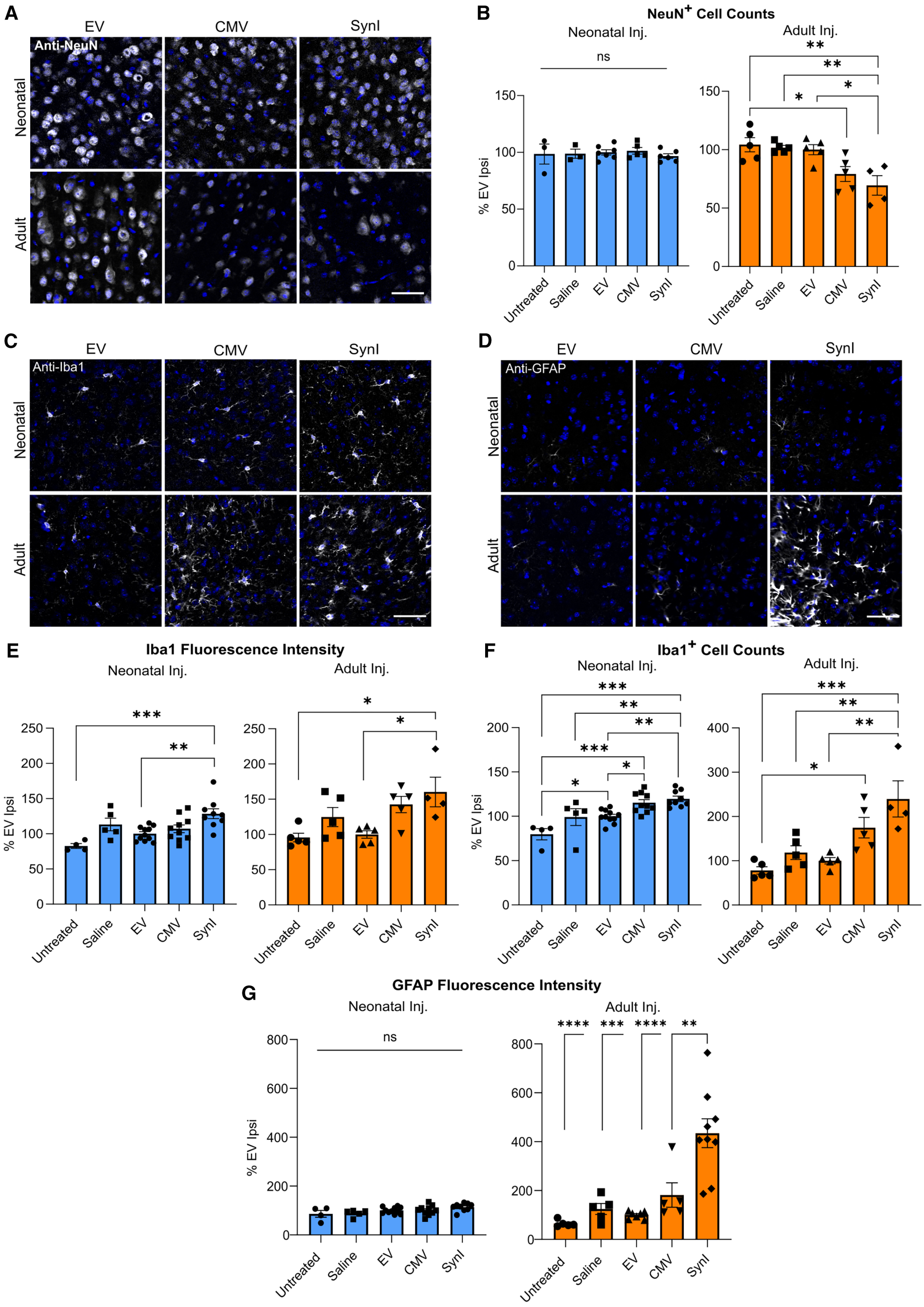

(legend on next page)

Analysis of neurotoxicity and neuroinflammation after AAV-Cas9 injections

The immunogenic effects of the two Cas9-HA-encoding AAVs were studied following injection into the neonatal and adult brain. Three control treatment groups were used in these analyses; one cohort of mice was administered AAV-EV (empty vector, Figure 1A) to control for the effects of the viral capsid and genome, another set was injected with sterile saline to assess the effects of the injection procedure alone, and the third group consisted of untreated, uninjected mice. To determine whether a Cas9-directed immune response had led to a loss of Cas9$^+$ neurons, we performed counts of neurons immunolabeled by anti-NeuN to quantify somatosensory cortical neuronal density (Figure 2A). Mice treated with the Cas9-encoding AAVs as neonates did not show a change in the number of NeuN$^+$ neurons in the somatosensory cortex relative to the control cohorts (Figures 2A and 2B). By contrast, infusion of AAV-SynI-Cas9 to the adult mouse brain resulted in a significant reduction of neuronal density compared with the three control treatment groups (−30.6% relative to EV, $p = 0.0111$; −32.3% relative to saline, $p = 0.0070$; −34.8% relative to untreated, $p = 0.0036$). AAV-CMV-Cas9 also induced a significant reduction in NeuN$^+$ neuronal density relative to untreated mice (−25.1%, $p = 0.0310$; Figures 2A and 2B). There was no loss of neurons in the EV or saline groups compared with uninjected mice (Figure 2B). The observed drops in neuronal density, coupled with the lack of Cas9-HA expression, indicate that infusions of Cas9-encoding AAVs into the adult, but not the neonatal mouse brain, induced a neurotoxic response.

Microglial and astrocyte activation in response to Cas9 expression was examined by IF staining using anti-Iba1 and anti-glial fibrillary acidic protein (GFAP), respectively (Figures 2C and 2D). Mice administered AAV-SynI-Cas9 as neonates displayed elevated Iba1 fluorescence intensity compared with AAV-EV (ipsilateral, +28.5%, $p = 0.0038$) and untreated mice (ipsilateral, +45.9%, $p = 0.0004$), but not saline-treated mice (ipsilateral, +15.3%, $p = 0.457$), as well as increased numbers of Iba1$^+$ microglia relative to all three control groups (ipsilateral, AAV-EV: +19.6%, $p = 0.0056$; saline: +20.5%, $p = 0.0261$; untreated: +39.8%, $p < 0.0001$) (Figures 2E and 2F). AAV-CMV-Cas9 also showed significantly elevated numbers of Iba1$^+$ microglia relative to AAV-EV-treated (ipsilateral, +15.2%, $p = 0.039$) and untreated (ipsilateral, +35.4%, $p = 0.0001$) mice, but not saline-treated mice (ipsilateral, +16.1%, $p = 0.110$) (Figure 2F). An increase in the number of Iba1$^+$ microglia in mice administered AAV-EV as neonates relative to age-matched untreated mice (ipsilateral, +20.2%, $p = 0.0409$) was observed, suggesting that the AAV capsid and/or vector genome was sufficient to elicit a detectable microglial response at this time point (Figure 2E). Furthermore, AAV-SynI-Cas9 treatment at PND 2 or 3 also resulted in an increase in Iba1 fluorescence intensity in the uninjected contralateral hemisphere (relative to EV: 30.6%, $p = 0.0020$; relative to untreated: +36.5%, $p = 0.007$), as well as elevated numbers of Iba1$^+$ microglia relative to untreated controls (28.2%, $p = 0.007$, Figures S3A and S3B). Mice treated with AAV-SynI-Cas9 as adults showed increases in both Iba1 fluorescence intensity (ipsilateral, relative to untreated: +64.6%, $p = 0.0122$; relative to EV: +60.3%, $p = 0.0206$) and number of Iba1$^+$ cells (ipsilateral, relative to untreated: +161.4%, $p = 0.0003$; relative to saline: +121.2%, $p = 0.0055$; relative to EV: +139.6%, $p = 0.0014$) compared with controls (Figures 2E and 2F). AAV-CMV-Cas9 showed a trend toward an increase in both metrics, although statistical significance was only observed in Iba1$^+$ cell counts relative to untreated controls (ipsilateral, +96.5%, $p = 0.0216$) (Figures 2E and 2F). No differences in Iba1 intensity or cell counts were detected between treatment groups in the contralateral hemispheres of adult treated mice (Figures S3A and S3B).

Analyses of the astrocyte marker GFAP by IF intensity in the neonatal cohort revealed no significant differences between treatment groups in the ipsilateral (Figure 2G) or the contralateral hemispheres (Figure S3C). However, AAV-SynI-Cas9 elicited a strong elevation in GFAP IF after injection into adult mice relative to the other four treatment groups (ipsilateral, relative to untreated: +369.5%, $p < 0.0001$; relative to saline: +310.0%; $p = 0.0002$; relative to EV: +334.5%, $p < 0.0001$; relative to CMV: +252.9%, $p = 0.003$) (Figure 2G). A potential explanation for the observed higher GFAP response to the SynI vector compared with the CMV vector in the adult injected animals (Figure 2G) is that the CMV-mediated expression in astrocytes facilitated the immune response, resulting in an earlier onset and subsequent resolution of the inflammatory response. Thus, the lower level of GFAP activation detected here at 4 weeks post-injection to CMV-Cas9 might be explained by the immune response "winding down." As observed in the neonatally injected mice, no change in GFAP expression was detected in the contralateral hemispheres in the adult treated mice (Figure S3C). These results imply that microglia became activated in response to Cas9 expression following AAV-Cas9 infusion at both the neonatal and adult stages. However, only AAV-SynI-Cas9 administered to the adult cerebral cortex resulted in the activation of astrocytes 4 weeks post-injection.

RT-qPCR analyses were conducted to examine selected cytokine expression profiles of the treated mice. Surprisingly, despite the

Figure 2. Neuronal loss and glial activation in AAV-Cas9-treated mice

(A) Representative immunostaining of NeuN$^+$ cells (gray) and DAPI (blue) in the ipsilateral somatosensory cerebral cortices of mice in each AAV treatment group. (B) Quantitative counts of NeuN$^+$ cells in the cerebral cortices of AAV-treated and control mice. (C) Representative immunostaining of Iba1$^+$ microglia (gray) and DAPI (blue) in the ipsilateral somatosensory cerebral cortices of mice in each AAV treatment group. (D) Representative immunostaining of GFAP$^+$ astrocytes (gray) and DAPI (blue) in AAV-treated mouse cerebral cortices. (E) Quantitative analyses of Iba1 fluorescence intensity in mice injected as neonates or adults. (F) Quantitative analyses of Iba1 cell counts in mice injected as neonates or adults. (G) Quantitative analyses of GFAP fluorescence intensity in mice injected as neonates or adults. Ipsi, ipsilateral. ns, not significant; *$p < 0.05$; **$p < 0.01$; ***$p < 0.001$; ****$p < 0.0001$ using Tukey's post hoc or Dunnett's multiple comparison test. Error bars indicate S.E.M. Scale bars, 50 μm.

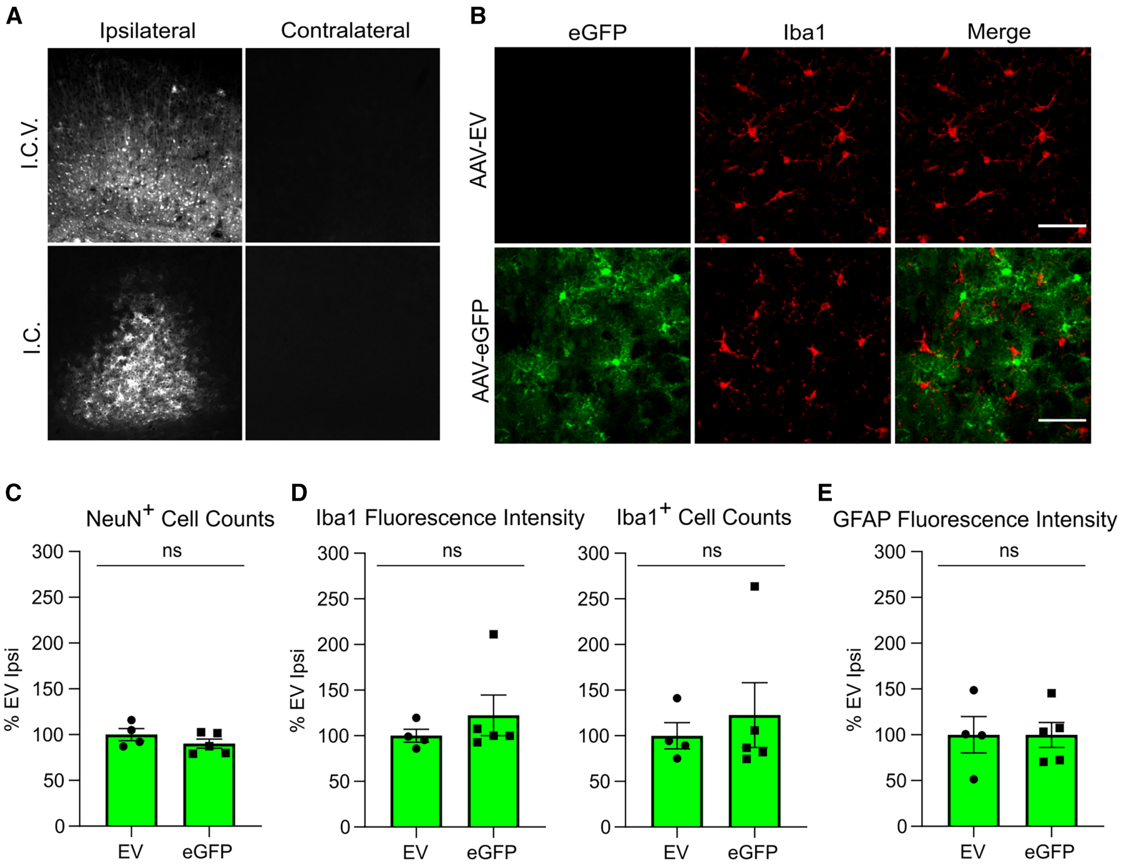

Figure 3. EGFP expression and absence of glial activation in the adult CNS after AAV-EGFP injection

(A) EGFP fluorescence in the ipsilateral and contralateral somatosensory cortices of mice injected with AAV-EGFP by i.c.v. or i.c. injection as adults. (B) Representative immunostaining of Iba1$^+$ microglia in the transduced the somatosensory cortex after i.c. injection. Scale bars, 50 μm. (C) Quantitative counts of NeuN$^+$ neurons in the somatosensory cortices of mice administered AAV-EV or AAV-EGFP by i.c. injection. (D) Quantitation of Iba1 immunofluorescence intensity and counts of Iba1$^+$ microglia in the somatosensory cortices of mice administered AAVs by i.c. injection. (E) Quantitation of GFAP immunofluorescence intensity in the somatosensory cortices of mice administered AAVs by i.c. injection. No statistically significant differences were detected by unpaired t tests. Ipsi, ipsilateral; ns, not significant. Error bars indicate S.E.M.

increases in glial activation detected by IF following AAV-Cas9 injections to the adult brain, the levels of pro-inflammatory (tumor necrosis factor [TNF]α, interleukin [IL]-1β, CCL2, interferon [IFN]γ) and anti-inflammatory (IL-10, transforming growth factor [TGF]-β) cytokines were not elevated in the cortices of adult mice treated with either of the Cas9-encoding AAVs relative to controls. The expression levels of these cytokines were also unchanged between experimental groups following neonatal injection (Figure S4). Altogether, these results show a stronger glial response to AAV-Cas9s following infusion to the adult brain than to the neonatal brain, while in both cases changes in cytokine expression levels were too low to be detected, or elevated levels had decreased to below the detection limit by the time of tissue sample collection.

Comparative effects of brain EGFP expression in the adult brain

Fluorescent proteins such as enhanced green fluorescent protein (EGFP), are derived from non-mammalian species (e.g., the hydrozoa *Aequorea victoria* in the case of EGFP) and are widely used as research tools to label cells *in vivo*. To ascertain whether expression of EGFP induced effects similar to Cas9 in the CNS, AAV-EGFP was administered into the adult mouse brain (Figure 1A). In contrast to Cas9, strong EGFP immunofluorescence was detected 1 month post-injection (Figures 3A and 3B) following both i.c.v. and i.c. injection. The numbers of NeuN$^+$ neurons (Figure 3C) and the activation levels of microglia (immunolabeled by anti-Iba-1, Figures 3B and 3D) and astrocytes (labeled by anti-GFAP, Figure 3E) in the ipsilateral somatosensory cortices were assessed by IF staining following i.c. injection. Injection of AAV-EGFP did not elicit differences relative to AAV-EV injection in the numbers of neurons nor in microglial and astrocyte activation (Figures 3C–3E), indicating that EGFP did not elicit neurotoxic or neuroinflammatory responses.

Assessment of adaptive cellular and humoral immunity in response to Cas9 expression in the CNS

It has been reported that transgenic non-self proteins may be eliminated by T cell responses when expressed in the CNS.[17] Therefore,

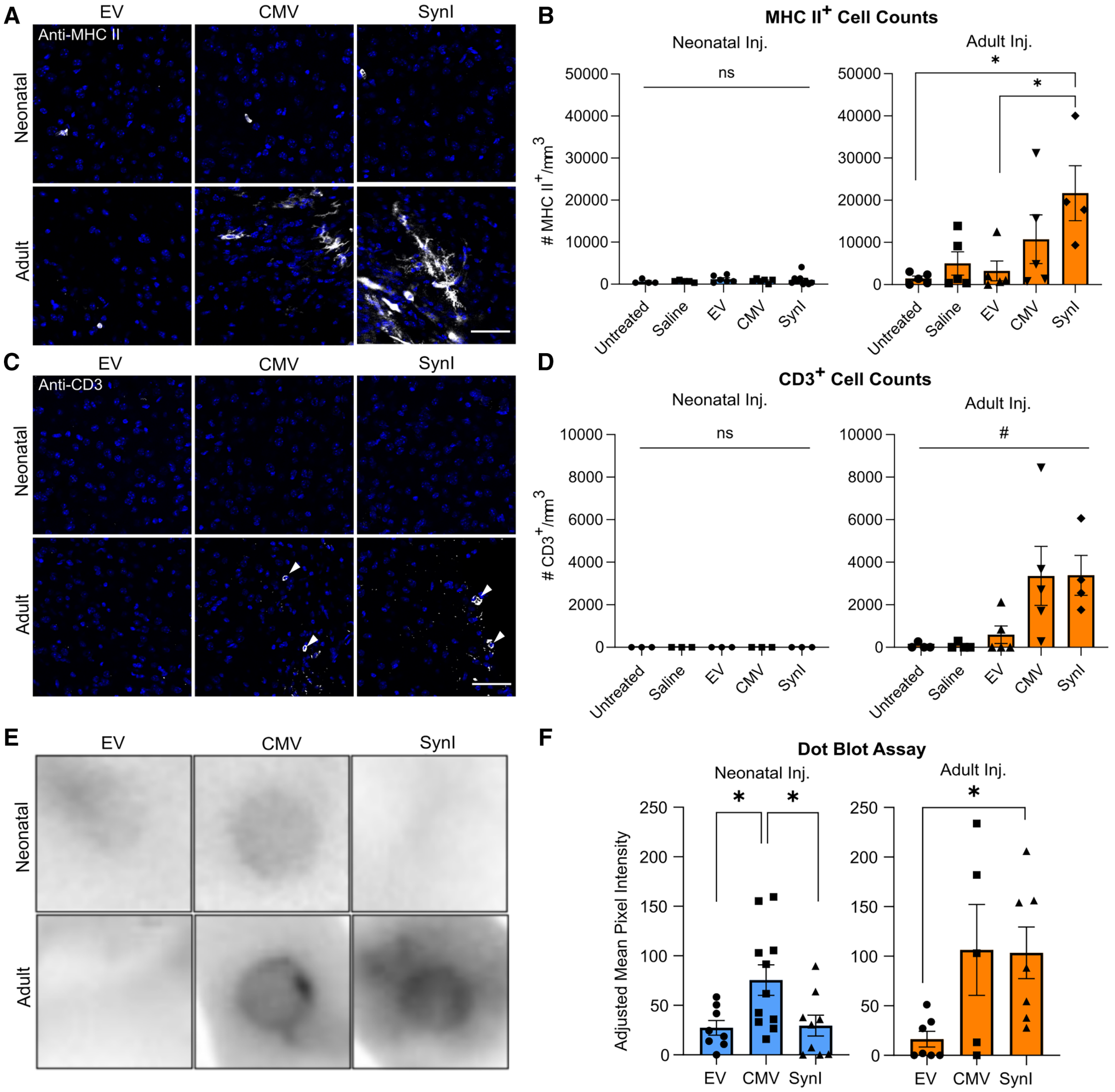

Figure 4. Induction of adaptive immunity in the CNS in neonatal vs. adult AAV-Cas9 injected mice

(A) Representative immunostaining of MHC II expression (gray) and DAPI (blue) in the ipsilateral somatosensory cerebral cortices of mice in each AAV treatment group. (B) Quantitative counts of MHC II-expressing cells. (C) Representative immunostaining of CD3 expression in the cerebral cortices of mice following AAV-EV, AAV-CMV-Cas9, and AAV-SynI-Cas9 treatment. (D) Quantitative counts of $CD3^+$ cells in the cerebral cortices of mice injected as neonates and as adults. (E) Representative dot blot assay results from sera of mice administered AAV-Cas9s as neonates or adults. (F) Quantitative results of serum dot blot assay; n = 5–11. Ipsi, ipsilateral; ns, not significant; #$p < 0.05$, one-way ANOVA; *$p < 0.05$, **$p < 0.01$, Tukey's post hoc or Dunnett's multiple comparisons tests. Error bars indicate S.E.M. Scale bars, 50 μm. All brain samples were collected 1 month post-injection.

we asked whether such a reaction might be responsible for the strong neurotoxic response we observed here following AAV-Cas9 injections. Microglia elicit adaptive T cell-mediated immune responses by antigen presentation via major histocompatibility complex II (MHC II).[26,27] IF staining using anti-MHC II revealed a significant increase in the number of MHC II-expressing cells in the ipsilateral somatosensory cortices of mice administered AAV-SynI-Cas9 (21,677 cells/mm^3) as adults relative to EV (3250 cells/mm^3), saline (5010 cells/mm^3), and untreated (1,422 cells/mm^3) controls (Figures 4A and 4B). Adult mice administered AAV-CMV-Cas9 showed a smaller, non-statistically significant increase in the number of MHC II-expressing cells (10,747 cells/mm^3) (Figures 4A and 4B). Infusions of AAV-SynI-Cas9 to the mature CNS resulted in 10.7% of $Iba1^+$ microglia co-expressing MHC II, whereas smaller proportions of $Iba1^+$ microglia co-expressed MHC II following treatment with AAV-CMV-Cas9 (4.6%), AAV-EV (1.7%), saline (3.0%), or in untreated mice (0.4%) (Figure S5). In the neonatally treated cohort, MHC II expression was found to be virtually absent (Figures 4A

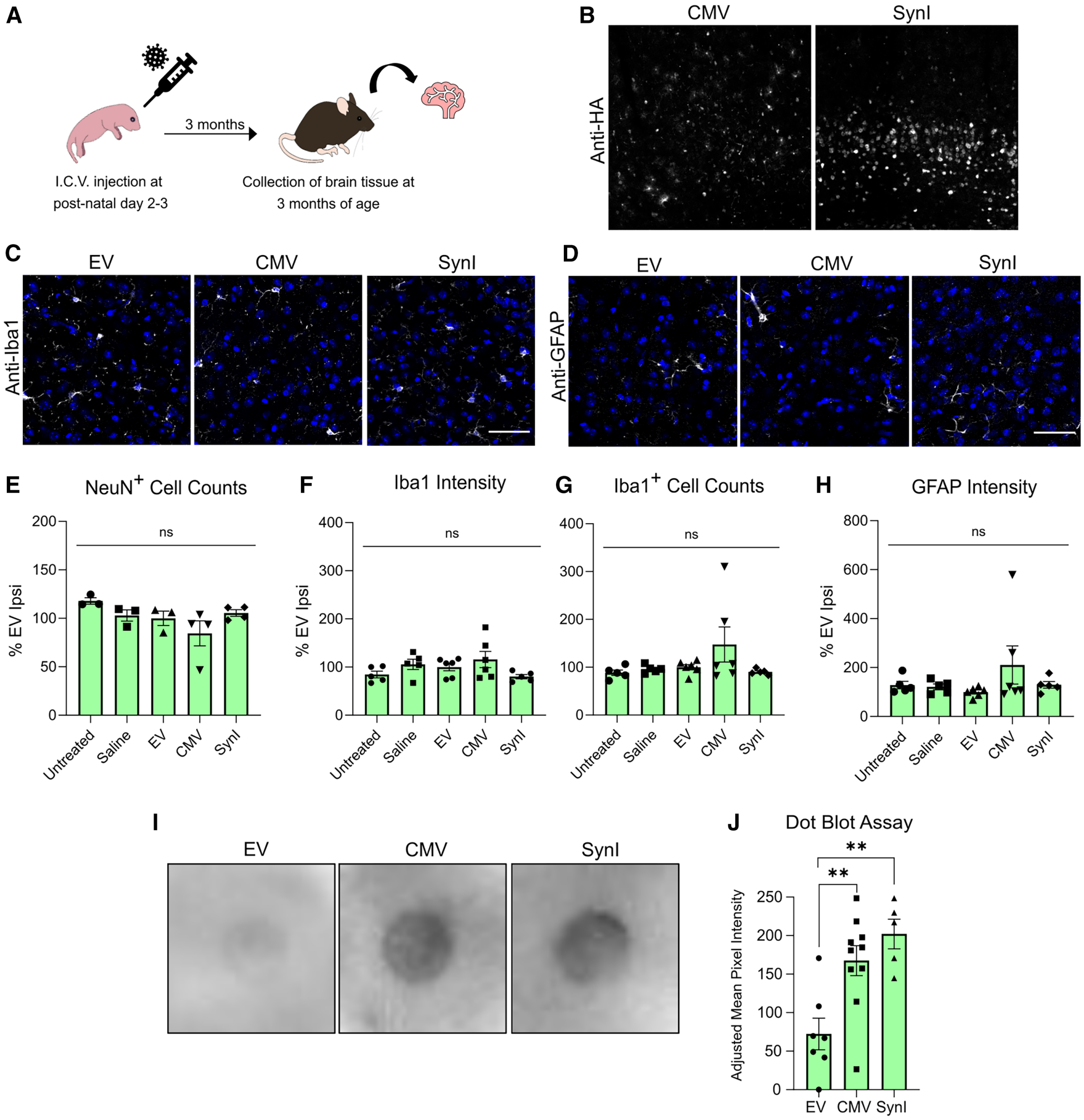

Figure 5. Analysis of immune activation in mice 3 months following neonatal AAV-Cas9 injection

(A) Schematic depiction of injection and collection timelines. (B) Representative immunostaining of Cas9-HA expression in the ipsilateral somatosensory cerebral cortices of 3-month-old mice. (C) Representative immunostaining of Iba1 (gray) and DAPI (blue) expression. Scale bar, 50 μm. (D) Representative immunostaining of GFAP (gray) and DAPI (blue) expression. Scale bar, 50 μm. (E) Quantitation of NeuN cell counts. (F) Quantitation of Iba1 fluorescence intensity. (G) Quantitation of Iba1 cell counts. (H) GFAP fluorescence intensity; N = 3–6 mice per cohort. (I) Representative examples of serum dot blot assay. (J) Quantitative results of serum dot blot assay; n = 5–10. ns, not significant; $^{**}p < 0.01$, Tukey's post hoc test. Error bars indicate S.E.M. All brain samples were collected 3 months after neonatal AAV injections.

and 4B); MHC II$^+$ cells were very sparse in the cerebral cortices (Figure 4B) and were largely not found to be co-positive for Iba1 (Figure S5), indicating that these cells were likely not microglia.

To ascertain whether T cell infiltration of the CNS occurred in response to AAV-Cas9, IF staining using the broad T cell marker anti-CD3 was performed. Mice treated as neonates showed no detectable CD3 response, while sparse numbers of CD3$^+$ T cells were detected at the injection site in adult mice administered AAV-SynI-Cas9 (3,386 cells/mm^3) and AAV-CMV-Cas9 (3,358 cells/mm^3) (Figures 4C and 4D). Even fewer CD3$^+$ T cells were detected in AAV-EV-treated (593 cells/mm^3) and saline-treated (73 cells/mm^3), and untreated (68 cells/mm^3) control mice (Figures 4C and 4D). Although a one-way ANOVA test indicated a significant difference between the means of the adult injected groups (p = 0.015), Tukey's multiple comparison test did not show significant

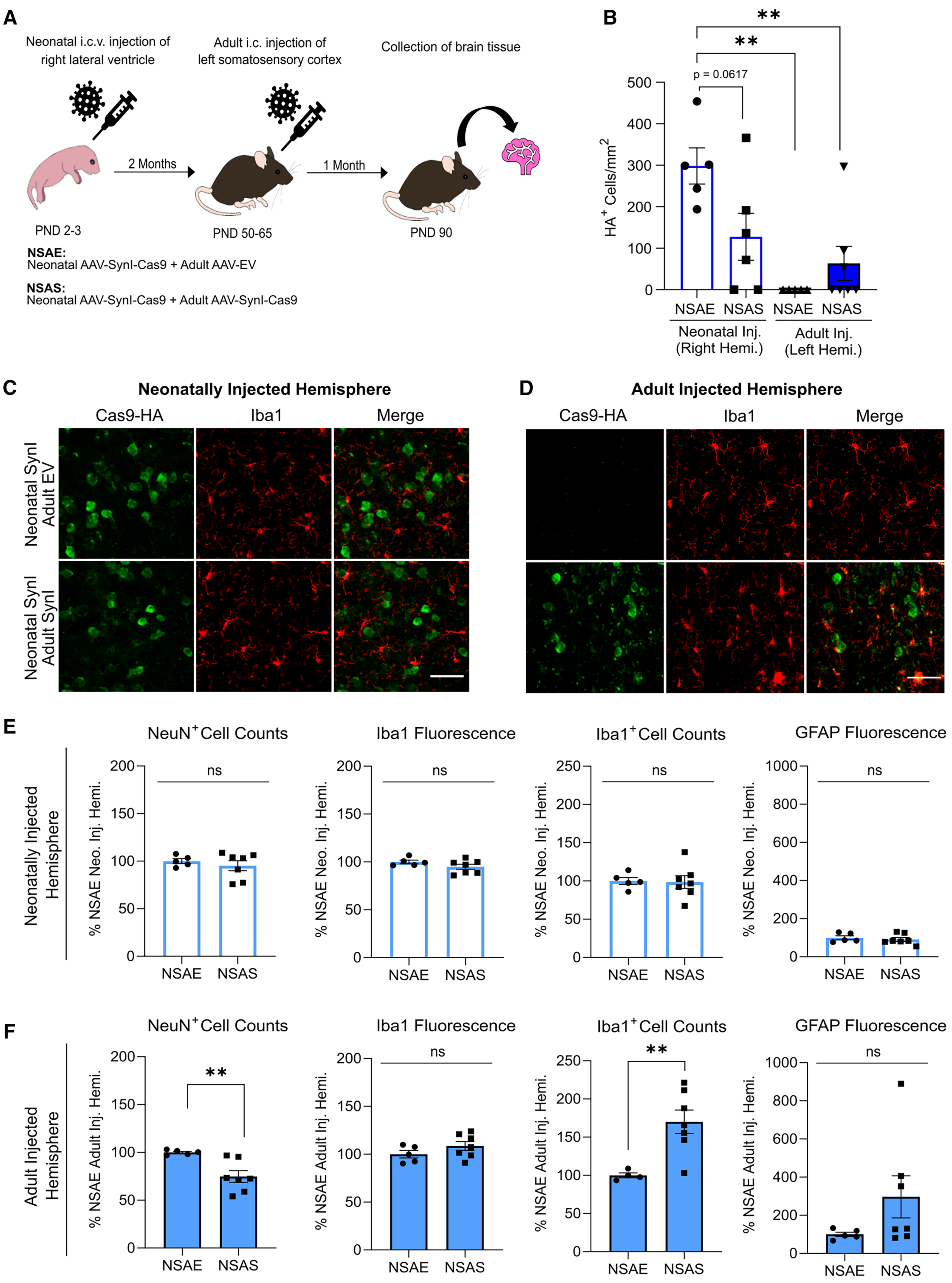

Figure 6. Redosing of AAV-SynI-Cas9 in adulthood following neonatal treatment

(A) Schematic of experimental design. The two treatment cohorts are defined as NSAE, neonatal AAV-SynI-Cas9 + adult AAV-EV, and NSAS, neonatal AAV-SynI-Cas9 + adult AAV-SynI-Cas9. (B) Results of Cas9-HA$^+$ cell counts in the somatosensory cortices. $^{**}p < 0.01$, Tukey's post hoc test. (C) Representative double immunolabeled images of Cas9-HA and Iba1 expression in the somatosensory cortices following neonatal injection. Scale bar, 50 μm. (D) Representative double immunolabeled images of

(legend continued on next page)

differences between individual groups, likely due to the high variability in the CMV and SynI-treated cohorts (Figure 4D). It has previously been shown that T cell infiltration of the CNS in response to the expression of foreign antigens is transient, with only low numbers of T cells present once the transgene is largely eliminated.[17] The lack of Cas9-HA expression following treatment of adult mice, and the accompanying decrease in neuronal density, suggests that the peak of T cell infiltration may have occurred prior to sample collection, and the scarce T cells detected corresponded to the tail end of the cellular immune response.

To determine whether the AAV-Cas9 vectors also induced an adaptive humoral immune response, the levels of Cas9-reactive immunoglobulin (Ig)G antibodies were quantified in the sera of injected mice. The results of dot-blotting experiments (Figure 4E) showed that neonatal infusion of AAV-CMV-Cas9 resulted in elevated levels of Cas9-reactive serum antibodies relative to both AAV-EV ($p = 0.0317$) and AAV-SynI-Cas9 ($p = 0.0343$), with no difference detected between the EV and SynI treatments ($p = 0.9923$, Figure 4F). In contrast, infusion of AAV-SynI-Cas9 to the adult brain resulted in pronounced Cas9-reactive IgG production relative to AAV-EV ($p = 0.041$). Adult injection of AAV-CMV-Cas9 also showed a trend toward increased anti-Cas9 IgG production relative to AAV-EV, but this was not statistically significant ($p = 0.29$). It is possible that preexisting anti-Cas9 antibodies contributed to some extent to the levels observed.[11,12] Altogether, these results indicate that treatment with AAV-Cas9 shortly after birth or in adults elicits a humoral antibody response, while the induction of a T cell response was only observed following adult brain administration.

Neurotoxicity and inflammation at 3 months post-injection in neonates

The results presented above describe immune responses at 1-month post-injection after early postnatal AAV-Cas9 treatment on PND 2 or 3; this developmental period corresponds to a time at which the mice are not yet fully mature. Therefore, to determine whether Cas9 expression and/or glial activation were sustained beyond 1 month post-injection, additional analyses were carried out in mice 3 months following injections on PND 2 or 3 (Figure 5A). IF staining of brain tissue with anti-HA revealed strong expression of Cas9-HA at this time point in the somatosensory cortices of mice injected with both AAV-SynI-Cas9 and AAV-CMV-Cas9, showing that an inflammatory response of sufficient strength to eliminate transgene-expressing cells was not triggered (Figure 5B). Accordingly, immunostaining with anti-NeuN, anti-Iba1, and anti-GFAP revealed no statistically significant differences between mice administered the Cas9-encoding constructs AAV-SynI and AAV-CMV and control mice (Figures 5C–5H). These findings demonstrate that in mice administered AAV-Cas9s as neonates, Cas9 expression is maintained and does not trigger neuroinflammatory responses even after full maturation of the nervous system and the immune system. The results of the dot blot analysis of the sera from this cohort showed the presence of Cas9-reactive IgG antibodies after treatment with both AAV-CMV-Cas9 ($p = 0.007$ relative to AAV-EV) and AAV-SynI-Cas9 ($p = 0.002$ relative to AAV-EV) (Figures 5I and 5J).

Assessment of the effects of redosing AAV-SynI-Cas9 during adulthood following prior neonatal treatment

To determine whether mice injected neonatally would show a blunted response to the effects of a second injection administered into the adult brain, the AAV-SynI-Cas9 vector was injected into PND 2–3 mice, and a second injection was made into the same mice at PND 50–65 (Figure 6A). Mice were administered AAV-SynI-Cas9 by injection of the right lateral ventricles and were readministered the same AAV into the left somatosensory cortex as adults (this treatment cohort is denoted as neonatal SynI-Cas9, adult SynI-Cas9 or NSAS). Expression of Cas9 and immune activation were compared with mice that received a dose of AAV-EV as adults (neonatal SynI-Cas9, adult empty vector, NSAE; Figure 6A).

In both Cas9 treatment cohorts, Cas9 expression was detected in the neonatally injected right somatosensory cortex by IF staining with anti-HA. Infusion of AAV-SynI-Cas9 during adulthood resulted in fewer Cas9$^+$ neurons being detected in the neonatally injected hemisphere than after adult treatment with AAV-EV (127 neurons/mm^2 and 298 neurons/mm^2, respectively), though this difference was not statistically significant (Tukey's post hoc test: $p = 0.062$; Figures 6B and 6C). Re-administration of AAV-SynI-Cas9 into the adult CNS following prior neonatal injection resulted in low levels of Cas9-HA expression in the left (adult injected) somatosensory cortex, with a mean density of 63 Cas9$^+$ neurons/mm^2 (relative to NSAE neonatally injected hemisphere, $p = 0.0054$; relative to NSAS neonatally injected hemisphere, $p = 0.689$). However, only three of seven mice analyzed in this treatment cohort showed Cas9 expression in the left hemisphere. No Cas9$^+$ neurons were detected in the adult injected hemisphere in the NSAE cohort (Figure 6B).

Analyses of neuronal cell density, microglial activation (Figures 6C and 6D), and astrocyte activation were conducted. Neuronal cell counts (NeuN$^+$) revealed no differences between treatment groups in the neonatally injected (right) somatosensory cortex (Figure 6E, left histogram). However, injections of AAV-SynI-Cas9 during adulthood resulted in a drop in neuronal density when compared with AAV-EV, showing that early postnatal treatment with AAV-SynI-Cas9 did not abrogate the neurotoxic effect of this construct (−25.3%, $p = 0.006$) (Figure 6F, left histogram). Immunostaining using anti-Iba1 and anti-GFAP showed no differences between the two

Cas9-HA and Iba1 expression in the somatosensory cortices following adult injection. Scale bar, 50 μm. (E) Quantitative results of NeuN$^+$ cell counts, Iba1 fluorescence intensity and counts, and GFAP fluorescence intensity in the right (neonatally injected) somatosensory cortex. (F) Quantitative analysis of NeuN$^+$ cell counts, Iba1 fluorescence intensity and counts, and GFAP fluorescence intensity in the left (adult injected) somatosensory cortex. ns, nonsignificant, $^{**}p < 0.01$, unpaired t test with Welch's correction. Error bars indicate S.E.M.

treatment groups in the right hemisphere (Figure 6E, middle and right histograms). Infusion of AAV-SynI-Cas9 during adulthood resulted in elevated numbers of microglia in the left somatosensory cortex relative to AAV-EV infusion (+70.2%, p = 0.004), although fluorescence intensity was unchanged between treatment groups (Figure 6F, middle histograms). The NSAS cohort also showed an increase in GFAP immunopositivity in the left somatosensory cortex, although this was not statistically significant (+196%, p = 0.126; Figure 6F, right histogram), suggesting that prior treatment with AAV-SynI-Cas9 blunted the astrocytic response. Taken together, these findings demonstrate that prior exposure to AAV-SynI-Cas9 shortly after birth lowers, but does not entirely eliminate, the neurotoxic and inflammatory effects of administering this construct to the adult CNS.

DISCUSSION

Our findings illustrate the presence of an inflammatory and neurotoxic response against Cas9 following AAV administration to the adult mouse CNS. Four weeks after infusions of AAV-Cas9 into the adult CNS, substantial activation of microglia and astrocytes was detected, along with increased expression of the antigen-presenting complex MHC II and increased $CD3^+$ T cells in the injected somatosensory cortex. The elevation of glial and immune markers was coupled with reduced neuronal density and undetectable Cas9 protein expression, implying that infusion of either AAV-SynI-Cas9 or AAV-CMV-Cas9 induced a cellular immune response to Cas9 that eliminated most or all of the transduced cells. The absence of detectable Cas9 protein occurred irrespective of whether the adult injections were made via the i.c.v. or i.c. routes of administration.

Compared with the adult injections, infusion of AAV-Cas9 into the early postnatal mouse CNS induced only a relatively mild and transient elevation of microglia, strong and sustained Cas9 protein expression in the cerebral cortex, and no detectable loss of neurons. Moreover, the absence of elevated MHC II expression and $CD3^+$ T cells in the transduced region indicated that the activated microglia did not trigger a T cell-driven adaptive immune response. Changes in activation of astrocytes were also not detected, and Cas9 expression was maintained for at least 3 months following a single neonatal injection. These findings indicate that AAV-Cas9 administration shortly following birth does not precipitate an antigen-directed immune response within the CNS and the elimination of transduced neurons, as observed when the same Cas9-expressing viral vectors are administered to the mature CNS. However, we also observed that both neonatal and adult AAV-Cas9 intra-CNS injections elicited the production of Cas9-reactive serum antibodies, demonstrating that neither treatment time point circumvented eliciting a humoral immune response.

In sharp contrast to Cas9, robust transgene expression and a lack of neuroinflammation in response to the jellyfish-derived EGFP protein was observed following AAV-EGFP injection into the adult mouse brain. A previous study reported only a mild immune response to EGFP in the C57BL/6 mouse strain,[28] in agreement with the results presented here. In contrast, an unexpected outcome from our study was the absence of detectable expression of Cas9 protein following a single AAV infusion to the adult brain. Because the form of Cas9 used here is derived from the commensal and pathogenic bacterium *S. aureus*, we posit that the mammalian immune system may be better equipped to detect and respond to the presence of Cas9 epitopes than to EGFP, resulting in a much stronger inflammatory response being triggered to Cas9. Cas9 proteins have previously been shown to be immunogenic in the CNS,[8,10,29] although these studies reported less potent inflammatory responses than seen here. It should be noted that we did not use germ-free mice, and therefore pre-existing immunological memory to *S. aureus* Cas9 might have enhanced the potency of the immune response following AAV-Cas9 injections to the adult animals.[15]

The immune responses to AAV-EV compared with the other two negative controls (uninjected and saline injected) were notably minimal; only one of the two measurements of microglial activation—$Iba1^+$ cell counts (but not Iba1 signal intensity)—was significantly elevated in comparison to the uninjected group in the neonatally injected mice (Figure 2F). Although other work has shown elevations in immune markers in response to AAV capsid components in the CNS,[30] our findings suggest that the AAV9 vector itself was not a potent activator of immunity 1 month post-injection. This could reflect another advantage of intra-cerebrospinal fluid drug administration compared with systemic (intravenous) administration.

The current standard approach for suppressing immune responses to AAV therapies in human patients is through the use of immunosuppressive drugs. Corticosteroids such as prednisolone, the mTOR inhibitor rapamycin, and mycophenolate mofetil are all commonly administered leading up to and following AAV injection.[31] These drugs have been effective at suppressing immune responses to the AAV viral particles. However, pharmacological inhibition of the immune system for prolonged periods is infeasible and unsafe, and protein expression from the AAV vector may last for many years following a single dose.[32] Other approaches to reduce the immunogenic risks of therapeutic transgenic proteins are therefore required. Several tactics to reduce the innate immunogenicity of Cas9 have been developed, including transiently expressing Cas9,[8,23,29,33,34] and the creation of engineered variants that remove or alter immunogenic epitopes from the protein.[35] However, these approaches still rely on the expression of polypeptides that are foreign to the immune system, and therefore may not entirely eliminate the risk of transgene-targeted inflammatory responses.

Exposure to non-self antigens shortly following birth has been shown to elicit immune tolerance to the polypeptide, resulting in attenuated immunity when the antigen is readministered again later.[36–39] Our observations revealed a modest protective effect of re-administering AAV-SynI-Cas9 during adulthood following an initial intra-CNS dose during early postnatal development (Figure 6). The development of antigen-specific $CD4^+$ T_{reg}s is thought to be critical for

inducing immune tolerance,[40–42] which relies on the recognition of the cognate antigen via MHC II. As expected, the AAV-SynI-Cas9 construct was highly neuron specific (Figure 1F). Neurons are not known to express MHC II, and therefore the use of the SynI promoter may have hampered the development of Cas9-reactive T_{reg}s.

Gene therapy employing Cas9 for DNA editing, or activation of gene expression, has shown promise in preclinical studies as candidate treatments for neurodevelopmental disorders.[43–46] Importantly, the precedent for CNS administration of AAVs to young children has been established by recent early-phase clinical trials examining the safety and efficacy of gene therapy for several neurodevelopmental diseases such as giant axon neuropathy (ClinicalTrials.gov Identifier: NCT02362438), Batten disease (NCT04737460, CLN7), Tay-Sachs disease (NCT04669535), and Canavan disease (NCT04998396). Intervention as early as possible after birth should be considered critical for the treatment of most neurodevelopmental disorders to minimize biochemical and physiological abnormalities resulting from disease-induced impairment of brain development. Moreover, AAV gene therapy studies conducted in mice have shown that very early postnatal AAV treatment results in superior diffusion in the brain after intra-cerebrospinal fluid injections, compared with delayed treatment.[47,48] Our findings illustrating relatively minimal immune activation in neonates after exposure to Cas9, and presumably a concomitant increase in safety and efficacy, provide yet another incentive for very early drug administration in CRISPR-Cas9 gene editing.

MATERIALS AND METHODS

Animal procedures

All protocols used in this study related to animal use were approved by the University of Toronto Animal Care Committee. C57Bl/6J wild-type mice were purchased from Jackson Laboratories and were housed on a 14:10-h light/dark cycle with *ad libitum* access to food and water. Both male and female mice were used in this study, with approximately equal numbers of both sexes included in each cohort.

AAVs

AAVs encoding *S. aureus* Cas9 with a hemagglutinin (HA) epitope tag and under the control of the SynI (AAV-SynI-Cas9; SL116042) or CMV (AAV-CMV-Cas9; SL116041) promoters were purchased from SignaGen Laboratories. Another AAV9, which was identical to the AAV-SynI-Cas9 but did not contain an HA tag, was custom synthesized by SignaGen Laboratories. An AAV encoding enhanced green fluorescent protein (EGFP) with a CAG promoter (AAV-EGFP) and a control AAV empty vector (AAV-EV) containing a CMV promoter, a woodchuck hepatitis posttranscriptional regulatory element, and a 3′ rabbit β-globin untranslated region—but no protein coding sequence—were obtained from the University of Pennsylvania Vector Core. AAVs from SignaGen were purified via double CsCl ultracentrifugation, with titers measured by real-time PCR (as is standard). Purity was assessed using an LAL gel-clot assay, and all AAVs used had 0.000 Unit/mL of endotoxin. University of Pennsylvania Vector Core purification was done by tangential flow filtration followed by iodixanol gradient purification and buffer exchange. The purity cutoff was less than 5 EU/mL of endotoxin. All AAVs used were capsid serotype 9. Schematics of these AAVs are shown in Figure 1A and the doses used are given in Figure 1B.

Intracerebroventricular AAV injections of neonatal mice

Custom injection needles were prepared using 30-gauge needles and high-performance liquid chromatography tubing, which were connected to a Quintessential stereotaxic injector (Stoetling). Neonatal mice on PND 2 or 3 were infused via the i.c.v. route (targeting the right lateral ventricle) with 1 μL of AAV at 1 mm anterior from the lambda, 1 mm lateral from the midline, and at a depth of 1.5 mm. The AAV solution was infused at a flow rate of 1 μL/min. The injection needle was held in place for 1 min following the infusion to prevent leakage after which the pups were immediately returned to their parent cage.

Intracerebroventricular and intracortical AAV injections of adult mice

Adult mice (PND 50–65) were anesthetized by continuous application of isoflurane gas. An amount of 20 mg/kg meloxicam was administered as an analgesic prior to the surgery. The scalp was shaved and cleaned by alternating wipes of 70% ethanol and 2% chlorhexidine gluconate. The mice were mounted on a stereotaxic frame, and an incision of the skin was made along the midline of the skull. A hole was drilled at the injection position through the skull, and the needle was carefully lowered to the appropriate depths. Intracerebroventricular injections were performed at 0.3 mm caudal from the bregma, 1.0 mm lateral from the midline, and at a depth of 2.5 mm. Intracortical injections were performed at 0.3 mm caudal from the bregma, 1.5 mm lateral at a depth of 1.0 mm, with the bevel of the needle oriented laterally away from the midline. Four microliters of AAV solution or saline was infused at a rate of 0.2 μL/min, and the needle was held in place for 1 min following the injection to prevent backflow. The same custom-made needle setup was used as for the neonatal injections. After careful removal of the needle, the skin incision was sutured, and the mice were allowed to recover on a heat source prior to return to the holding room. Daily health checks were performed for 3 days following the procedure, with no adverse events being noted. Follow-up injections of 20 mg/kg of meloxicam were performed 24 h and 48 h following the surgery.

IF staining

Mice were anesthetized with a mixture of ketamine and xylazine by intraperitoneal injection, and transcardially perfused with 40 mL of ice-cold 1× phosphate-buffered saline (PBS), followed by 50 mL of ice-cold 4% paraformaldehyde in 1× PBS. The brains were removed and post-fixed by submersion in 4% paraformaldehyde solution for 24 h, followed by cryoprotection in 30% sucrose in 1× PBS for an additional 24 h. Brains were frozen in optimal cutting temperature (OCT) mounting medium (Fisher Healthcare), and 20- to 30-μm coronal sections were cut using a Leica CM3050S cryostat.

Unless otherwise specified, all steps were performed at room temperature. IF staining was performed by washing sections twice with 1× PBS, followed by permeabilization of tissue with 1 mL of 1% Triton X-100 in 1× PBS for 30 min. This solution was removed by washing with 1× PBS, and sections were incubated in blocking buffer (5% donkey serum, 5% bovine serum albumin in 1× PBS) for 1 hour. Sections were then incubated in primary antibodies diluted as appropriate in blocking buffer overnight at 4°C. The primary antibody solution was removed by three successive washes with 1× PBS, and the samples were incubated in secondary antibodies diluted in 5% donkey serum and 5 μg/mL of DAPI in 1× PBS. Following three washes, the sections were mounted on glass slides, dried, and covered with Prolong Gold-Antifade solution. The slides were stored in the dark at 4°C until image acquisition.

To detect Cas9, Cas9-HA, and EGFP expression in the brain, serial sections at intervals of ∼300 μm surrounding the injection site were analyzed. For Cas9 and Cas9-HA, IF staining was performed using rabbit anti-Cas9 (1:500, AB203933, Abcam) or rat anti-HA (1:750, ROAHAHA, Roche) primary antibody and anti-rabbit Alexa Fluor 594 (1:2,000, A21207, Invitrogen) or anti-rat Alexa Fluor 594 (1:2,000, 112-586-003, Jackson ImmunoResearch) secondary antibody. Images were acquired using the tiling feature on a Bio-Rad Cytation5 slide scanner at 4× magnification.

For IF analyses of immune markers, sections from Cas9-encoding AAV-treated mice were selected from within ∼300 μm of the injection site (in mice with no detectable Cas9-HA expression), or Cas9 from sections showing the highest level of transgene expression (as assessed by staining with anti-HA or EGFP fluorescence). Control sections from untreated, saline-, and AAV-EV-treated mice were position-matched based on anatomical features. Images were acquired using a Zeiss LSM 700 confocal microscope at 10× or 20× magnification. The laser power, gain, and pinhole size were kept constant between all images taken for an individual experiment. The sizes of the acquisition areas ranged from approximately 0.3 mm^2–0.8 mm^2. Fluorescence intensity analyses and cell counting were performed using ImageJ Fiji. Representative images were linearly adjusted in ImageJ Fiji for presentation. Antibodies used included mouse anti-NeuN (1:2,000, MAB377, EMD Millipore), rabbit anti-NeuN (1:5,000, ab177487, Abcam), rabbit anti-Iba1 (1:1,500, 019–19741, WAKO), rabbit anti-GFAP (1:2,000, D1F4Q, Cell Signaling Technology), rat anti-MHC II (1:1,500, MABF33, EMD Millipore), and rabbit anti-CD3 (1:200, C7930, Millipore-Sigma). Secondary antibodies used were anti-rabbit Alexa Fluor 488 (1:2000, A32731, Invitrogen), anti-rabbit Alexa Fluor 594 (1:2,000, A21207, Invitrogen), anti-mouse Alexa Fluor 488 (1:2,000, A11029, Invitrogen), anti-rat Alexa Fluor 594 (1:2,000, 112-586-003, Jackson ImmunoResearch), and anti-rat Alexa Fluor 647 (1:2,000, A21247, Invitrogen).

Real-time quantitative polymerase chain reaction (RT-qPCR)

Mice were anesthetized by intraperitoneal injection of ketamine/xylazine and transcardially perfused with 40 mL of ice-cold, sterile, RNase-free 1× PBS. The brains were removed, and the ipsilateral cerebral cortices (∼2 mm surrounding the injection position) were dissected from the rest of the brain. Cerebral cortical tissues were flash frozen on dry ice and stored at −80°C until RNA extraction. Tissues were homogenized and RNA was isolated using the Qiagen Mini RNeasy kit per the manufacturer's instructions. Extracted RNA concentration and purity were measured using a NanoDrop One (Thermo Scientific). Two micrograms of the RNA sample was reverse transcribed to cDNA using the SuperScript IV kit (Invitrogen) per the manufacturer's instructions. Quantitative PCR (qPCR) was performed using the PowerTrack SYBR Green kit (Applied Biosystems) along with gene-specific primer pairs (listed in Table S1); the reaction was run on a CFX 384 (Bio-Rad) thermocycler. Six nanograms (for SaCas9 quantification) or 25 ng (for cytokine quantification) of cDNA was run in triplicate for each reaction. *Pgk1* and *Ppia* were used as housekeeping genes, with the geometric means of the two genes being used for normalization.[49] The fold changes in gene expression in each treatment group were compared with the EV-treated group (cytokine expression) or the neonatal AAV-Cas9-treated group (Cas9 expression) using the delta-delta threshold method ($2^{-\Delta\Delta Ct}$). Positive controls for cytokine expression consisted of RNA extracted from cortical tissue of mice administered 2.0 mg/kg of lipopolysaccharide (LPS) (14011S, Cell Signaling Technology) by intraperitoneal injection 24–72 h prior to sample collection. For quantification of Cas9 expression, a DNase I digestion step was performed following RNA isolation using the manufacturer's protocol (M0303, New England Biolabs).

Dot blot assay

Blood samples were collected from mice by cardiac puncture immediately prior to sample collection for IF staining. Blood was added to microvette serum collection tubes (20.1343.100, Sarstedt) and allowed to clot for a minimum of 15 min at room temperature. The samples were then centrifuged at 10,000 × *g* for 5 min, and the serum layer was carefully removed by pipetting, aliquoted, flash frozen on dry ice, and stored at −80°C until analysis.

Two microliters of recombinant *S. aureus* Cas9 protein (60 ng total, K044, ABM) was blotted onto nitrocellulose membrane. After drying, the membranes were blocked for 1 hour using 5% bovine serum albumin in 1× Tris-buffered saline with 0.1% Tween 20 (TBS-T). Serum samples were diluted 1:10 in blocking buffer, and 20 μL of the diluted sera was pipetted onto the membranes, which were wrapped in parafilm and incubated for 30 min. The membranes were washed three times with 1× TBS-T and incubated with horseradish peroxidase-conjugated anti-mouse IgG secondary antibody diluted in blocking buffer (1:5,000, 115-035-003, Jackson ImmunoResearch) for 30 min. Three more washes with 1× TBS-T were performed, followed by a final wash with 1× Tris-buffered saline without Tween 20. Imaging was performed using the SuperSignal West Pico PLUS Chemiluminescent Substrate Kit (Thermo Scientific) per the manufacturer's instructions. Primary mouse anti-Cas9 antibody was used as a positive control (1:1,000, A01951-40, GenScript). We observed highly variable background signals in this assay that prevented us from performing direct

mean pixel intensity or densitometry quantification (this was likely a result of nonspecific binding of serum factors to the blot membrane, as we used a very low sample dilution). To account for this, image quantification was performed in ImageJ Fiji by gating the background for each individual dot image and identifying immunopositive pixels above the threshold. Positive pixels were converted to a binary value of 255, and negative pixels to 0. The mean values of the binary pixels were then measured within a standardized area surrounding the blotted dot.

Statistics

When comparing two means, statistical significance was assessed using an unpaired t test. Differences between three or more means were detected by one-way ANOVA, followed by Tukey's post hoc test. When variances between treatment groups were significantly different, as determined by an F-test or Brown-Forsythe test, Welch's correction was applied to unpaired t tests when comparing two treatment groups, and Welch's ANOVA followed by Dunnett's multiple comparison test was used when comparing three or more treatment groups. Outlier values were excluded from analyses if they attained a threshold of $p < 0.01$ by Grubb's test. All histograms are presented as the mean ± standard error of the mean. All statistical analyses were conducted using GraphPad Prism 10.

DATA AND CODE AVAILABILITY

All data needed to evaluate the conclusions in the paper are present in the paper and/or the supplemental information.

ACKNOWLEDGMENTS

We thank Drs. Hayes Wong, Yosuke Niibori, and Alexander Hooper for technical advice and helpful comments on the manuscript. We thank the Center for Pharmaceutical Oncology in the Leslie Dan Faculty of Pharmacy, University of Toronto and the Advanced Optical Microscopy Facility for instrumentation support. This research was supported by an operating grant to D.R.H. from the Canadian Institutes of Health Research (PJT 183963).

AUTHOR CONTRIBUTIONS

The project was conceived by D.R.H. and R.D.-K. The laboratory experiments were carried out by R.D.-K. The work was supervised by D.R.H. All drafts of the manuscript were written by both R.D.-K. and D.R.H.

DECLARATION OF INTERESTS

The authors declare no competing interests.

SUPPLEMENTAL INFORMATION

Supplemental information can be found online at https://doi.org/10.1016/j.omtm.2025.101536.

REFERENCES

1. Shen, W., Liu, S., and Ou, L. (2022). rAAV immunogenicity, toxicity, and durability in 255 clinical trials: A meta-analysis. Front. Immunol. *13*, 1001263. https://doi.org/10.3389/fimmu.2022.1001263.1.
2. Kachanov, A., Kostyusheva, A., Brezgin, S., Karandashov, I., Ponomareva, N., Tikhonov, A., Lukashev, A., Pokrovsky, V., Zamyatnin, A.A., Jr., Parodi, A., et al. (2024). The menace of severe adverse events and deaths associated with viral gene therapy and its potential solution. Med. Res. Rev. *44*, 2112–2193. https://doi.org/10.1002/med.22036.
3. Scott, D.W., and Pratt, K.P. (2019). Factor VIII: Perspectives on Immunogenicity and Tolerogenic Strategies. Front. Immunol. *10*, 3078. https://doi.org/10.3389/fimmu.2019.03078.
4. Abdelnour, S.A., Xie, L., Hassanin, A.A., Zuo, E., and Lu, Y. (2021). The Potential of CRISPR/Cas9 Gene Editing as a Treatment Strategy for Inherited Diseases. Front. Cell Dev. Biol. *9*, 699597. https://doi.org/10.3389/fcell.2021.699597.
5. Hana, S., Peterson, M., McLaughlin, H., Marshall, E., Fabian, A.J., McKissick, O., Koszka, K., Marsh, G., Craft, M., Xu, S., et al. (2021). Highly efficient neuronal gene knockout in vivo by CRISPR-Cas9 via neonatal intracerebroventricular injection of AAV in mice. Gene Ther. *28*, 646–658. https://doi.org/10.1038/s41434-021-00224-2.
6. Godbout, K., and Tremblay, J.P. (2023). Prime Editing for Human Gene Therapy: Where Are We Now? Cells *12*, 536. https://doi.org/10.3390/cells12040536.
7. Chew, W.L., Tabebordbar, M., Cheng, J.K.W., Mali, P., Wu, E.Y., Ng, A.H.M., Zhu, K., Wagers, A.J., and Church, G.M. (2016). A multi-functional AAV-CRISPR-Cas9 and its host response. Nat. Methods *13*, 868–874. https://doi.org/10.1038/nmeth.3993.
8. Yang, S., Li, S., and Li, X.-J. (2018). Shortening the Half-Life of Cas9 Maintains Its Gene Editing Ability and Reduces Neuronal Toxicity. Cell Rep. *25*, 2653–2659.e3. https://doi.org/10.1016/j.celrep.2018.11.019.
9. Hakim, C.H., Kumar, S.R.P., Pérez-López, D.O., Wasala, N.B., Zhang, D., Yue, Y., Teixeira, J., Pan, X., Zhang, K., Million, E.D., et al. (2021). Cas9-specific immune responses compromise local and systemic AAV CRISPR therapy in multiple dystrophic canine models. Nat. Commun. *12*, 6769. https://doi.org/10.1038/s41467-021-26830-7.
10. Gumusgoz, E., Kasiri, S., Youssef, I., Verma, M., Chopra, R., Villarreal Acha, D., Wu, J., Marriam, U., Alao, E., Chen, X., et al. (2025). Focused ultrasound widely broadens AAV-delivered Cas9 distribution and activity. Gene Ther. *32*, 237–245. https://doi.org/10.1038/s41434-025-00517-w.
11. Simhadri, V.L., McGill, J., McMahon, S., Wang, J., Jiang, H., and Sauna, Z.E. (2018). Prevalence of Pre-existing Antibodies to CRISPR-Associated Nuclease Cas9 in the USA Population. Mol. Ther. Methods Clin. Dev. *10*, 105–112. https://doi.org/10.1016/j.omtm.2018.06.006.
12. Charlesworth, C.T., Deshpande, P.S., Dever, D.P., Camarena, J., Lemgart, V.T., Cromer, M.K., Vakulskas, C.A., Collingwood, M.A., Zhang, L., Bode, N.M., et al. (2019). Identification of preexisting adaptive immunity to Cas9 proteins in humans. Nat. Med. *25*, 249–254. https://doi.org/10.1038/s41591-018-0326-x.
13. Wagner, D.L., Amini, L., Wendering, D.J., Burkhardt, L.-M., Akyüz, L., Reinke, P., Volk, H.-D., and Schmueck-Henneresse, M. (2019). High prevalence of Streptococcus pyogenes Cas9-reactive T cells within the adult human population. Nat. Med. *25*, 242–248. https://doi.org/10.1038/s41591-018-0204-6.
14. Arnson, B., Kang, H.R., Brooks, E.D., Gheorghiu, D., Ilich, E., Courtney, D., Everitt, J.I., Cullen, B.R., and Koeberl, D.D. (2023). Genome editing using Staphylococcus aureus Cas9 in a canine model of glycogen storage disease Ia. Mol. Ther. Methods Clin. Dev. *29*, 108–119. https://doi.org/10.1016/j.omtm.2023.03.001.
15. Li, A., Tanner, M.R., Lee, C.M., Hurley, A.E., De Giorgi, M., Jarrett, K.E., Davis, T.H., Doerfler, A.M., Bao, G., Beeton, C., and Lagor, W.R. (2020). AAV-CRISPR Gene Editing Is Negated by Pre-existing Immunity to Cas9. Mol. Ther. *28*, 1432–1441. https://doi.org/10.1016/j.ymthe.2020.04.017.
16. Hadaczek, P., Forsayeth, J., Mirek, H., Munson, K., Bringas, J., Pivirotto, P., McBride, J. L., Davidson, B.L., and Bankiewicz, K.S. (2009). Transduction of nonhuman primate brain with adeno-associated virus serotype 1: vector trafficking and immune response. Hum. Gene Ther. *20*, 225–237. https://doi.org/10.1089/hum.2008.151.
17. Ciesielska, A., Hadaczek, P., Mittermeyer, G., Zhou, S., Wright, J.F., Bankiewicz, K. S., and Forsayeth, J. (2013). Cerebral Infusion of AAV9 Vector-encoding Non-self Proteins Can Elicit Cell-mediated Immune Responses. Mol. Ther. *21*, 158–166. https://doi.org/10.1038/mt.2012.167.
18. Samaranch, L., Sebastian, W.S., Kells, A.P., Salegio, E.A., Heller, G., Bringas, J.R., Pivirotto, P., DeArmond, S., Forsayeth, J., and Bankiewicz, K.S. (2014). AAV9-mediated Expression of a Non-self Protein in Nonhuman Primate Central Nervous System Triggers Widespread Neuroinflammation Driven by Antigen-presenting Cell Transduction. Mol. Ther. *22*, 329–337. https://doi.org/10.1038/mt.2013.266.
19. Yang, C., Hao, F., He, J., Lu, T., Klein, R.L., Zhao, L.-R., and Duan, W.-M. (2016). Sequential Adeno-Associated Viral Vector Serotype 9-Green Fluorescent Protein

Gene Transfer Causes Massive Inflammation and Intense Immune Response in Rat Striatum. Hum. Gene Ther. *27*, 528–543. https://doi.org/10.1089/hum.2015.083.

20. Kofoed, R.H., Heinen, S., Silburt, J., Dubey, S., Dibia, C.L., Maes, M., Simpson, E.M., Hynynen, K., and Aubert, I. (2021). Transgene distribution and immune response after ultrasound delivery of rAAV9 and PHP.B to the brain in a mouse model of amyloidosis. Mol. Ther. Methods Clin. Dev. *23*, 390–405. https://doi.org/10.1016/j.omtm.2021.10.001.

21. Hordeaux, J., Dubreil, L., Deniaud, J., Iacobelli, F., Moreau, S., Ledevin, M., Le Guiner, C., Blouin, V., Le Duff, J., Mendes-Madeira, A., et al. (2015). Efficient central nervous system AAVrh10-mediated intrathecal gene transfer in adult and neonate rats. Gene Ther. *22*, 316–324. https://doi.org/10.1038/gt.2014.121.

22. Nelson, C.E., Wu, Y., Gemberling, M.P., Oliver, M.L., Waller, M.A., Bohning, J.D., Robinson-Hamm, J.N., Bulaklak, K., Castellanos Rivera, R.M., Collier, J.H., et al. (2019). Long-term Evaluation of AAV-CRISPR Genome Editing for Duchenne Muscular Dystrophy. Nat. Med. *25*, 427–432. https://doi.org/10.1038/s41591-019-0344-3.

23. Ibraheim, R., Tai, P.W.L., Mir, A., Javeed, N., Wang, J., Rodríguez, T.C., Namkung, S., Nelson, S., Khokhar, E.S., Mintzer, E., et al. (2021). Self-inactivating, all-in-one AAV vectors for precision Cas9 genome editing via homology-directed repair in vivo. Nat. Commun. *12*, 6267. https://doi.org/10.1038/s41467-021-26518-y.

24. Semple, B.D., Blomgren, K., Gimlin, K., Ferriero, D.M., and Noble-Haeusslein, L.J. (2013). Brain development in rodents and humans: Identifying benchmarks of maturation and vulnerability to injury across species. Prog. Neurobiol. *106–107*, 1–16. https://doi.org/10.1016/j.pneurobio.2013.04.001.

25. Thornlow, D.N., Macintyre, A.N., Oguin, T.H., Karlsson, A.B., Stover, E.L., Lynch, H.E., Sempowski, G.D., and Schmidt, A.G. (2021). Altering the Immunogenicity of Hemagglutinin Immunogens by Hyperglycosylation and Disulfide Stabilization. Front. Immunol. *12*, 737973. https://doi.org/10.3389/fimmu.2021.737973.

26. Aloisi, F., Ria, F., and Adorini, L. (2000). Regulation of T-cell responses by CNS antigen-presenting cells: different roles for microglia and astrocytes. Immunol. Today *21*, 141–147. https://doi.org/10.1016/s0167-5699(99)01512-1.

27. Graeber, M.B., Li, W., and Rodriguez, M.L. (2011). Role of microglia in CNS inflammation. FEBS Lett. *585*, 3798–3805. https://doi.org/10.1016/j.febslet.2011.08.033.

28. Skelton, D., Satake, N., and Kohn, D.B. (2001). The enhanced green fluorescent protein (eGFP) is minimally immunogenic in C57BL/6 mice. Gene Ther. *8*, 1813–1814. https://doi.org/10.1038/sj.gt.3301586.

29. Stahl, E.C., Sabo, J.K., Kang, M.H., Allen, R., Applegate, E., Kim, S.E., Kwon, Y., Seth, A., Lemus, N., Salinas-Rios, V., et al. (2023). Genome editing in the mouse brain with minimally immunogenic Cas9 RNPs. Mol. Ther. *31*, 2422–2438. https://doi.org/10.1016/j.ymthe.2023.06.019.

30. Suriano, C.M., Kumar, N., Verpeut, J.L., Ma, J., Jung, C., Dunn, C.E., Carvajal, B.V., Nguyen, A.V., and Boulanger, L.M. (2024). An innate immune response to adeno-associated virus genomes decreases cortical dendritic complexity and disrupts synaptic transmission. Mol. Ther. *32*, 1721–1738. https://doi.org/10.1016/j.ymthe.2024.03.036.

31. Prasad, S., Dimmock, D.P., Greenberg, B., Walia, J.S., Sadhu, C., Tavakkoli, F., and Lipshutz, G.S. (2022). Immune Responses and Immunosuppressive Strategies for Adeno-Associated Virus-Based Gene Therapy for Treatment of Central Nervous System Disorders: Current Knowledge and Approaches. Hum. Gene Ther. *33*, 1228–1245. https://doi.org/10.1089/hum.2022.138.

32. Buchlis, G., Podsakoff, G.M., Radu, A., Hawk, S.M., Flake, A.W., Mingozzi, F., and High, K.A. (2012). Factor IX expression in skeletal muscle of a severe hemophilia B patient 10 years after AAV-mediated gene transfer. Blood *119*, 3038–3041. https://doi.org/10.1182/blood-2011-09-382317.

33. Merienne, N., Vachey, G., de Longprez, L., Meunier, C., Zimmer, V., Perriard, G., Canales, M., Mathias, A., Herrgott, L., Beltraminelli, T., et al. (2017). The Self-Inactivating KamiCas9 System for the Editing of CNS Disease Genes. Cell Rep. *20*, 2980–2991. https://doi.org/10.1016/j.celrep.2017.08.075.

34. Li, A., Lee, C.M., Hurley, A.E., Jarrett, K.E., De Giorgi, M., Lu, W., Balderrama, K.S., Doerfler, A.M., Deshmukh, H., Ray, A., et al. (2019). A Self-Deleting AAV-CRISPR System for In Vivo Genome Editing. Mol. Ther. Methods Clin. Dev. *12*, 111–122. https://doi.org/10.1016/j.omtm.2018.11.009.

35. Ferdosi, S.R., Ewaisha, R., Moghadam, F., Krishna, S., Park, J.G., Ebrahimkhani, M. R., Kiani, S., and Anderson, K.S. (2019). Multifunctional CRISPR-Cas9 with engineered immunosilenced human T cell epitopes. Nat. Commun. *10*, 1842. https://doi.org/10.1038/s41467-019-09693-x.

36. Hinderer, C., Bell, P., Louboutin, J.-P., Zhu, Y., Yu, H., Lin, G., Choa, R., Gurda, B.L., Bagel, J., O'Donnell, P., et al. (2015). Neonatal Systemic AAV Induces Tolerance to CNS Gene Therapy in MPS I Dogs and Nonhuman Primates. Mol. Ther. *23*, 1298–1307. https://doi.org/10.1038/mt.2015.99.

37. Piccand, M., Bessa, J., Schick, E., Senn, C., Bourquin, C., and Richter, W.F. (2016). Neonatal Immune Tolerance Induction to Allow Long-Term Studies With an Immunogenic Therapeutic Monoclonal Antibody in Mice. AAPS J. *18*, 354–361. https://doi.org/10.1208/s12248-015-9850-5.

38. Tai, D.S., Hu, C., Lee, C.C.I., Martinez, M., Cantero, G., Kim, E.H., Tarantal, A.F., and Lipshutz, G.S. (2015). Development of Operational Immunologic Tolerance with Neonatal Gene Transfer in Nonhuman Primates: Preliminary Studies. Gene Ther. *22*, 923–930. https://doi.org/10.1038/gt.2015.65.

39. Hordeaux, J., Hinderer, C., Buza, E.L., Louboutin, J.-P., Jahan, T., Bell, P., Chichester, J.A., Tarantal, A.F., and Wilson, J.M. (2019). Safe and Sustained Expression of Human Iduronidase After Intrathecal Administration of Adeno-Associated Virus Serotype 9 in Infant Rhesus Monkeys. Hum. Gene Ther. *30*, 957–966. https://doi.org/10.1089/hum.2019.012.

40. Mold, J.E., Michaëlsson, J., Burt, T.D., Muench, M.O., Beckerman, K.P., Busch, M.P., Lee, T.-H., Nixon, D.F., and McCune, J.M. (2008). Maternal Alloantigens Promote the Development of Tolerogenic Fetal Regulatory T Cells in Utero. Science *322*, 1562–1565. https://doi.org/10.1126/science.1164511.

41. Simon, A.K., Hollander, G.A., and McMichael, A. (2015). Evolution of the immune system in humans from infancy to old age. Proc. Biol. Sci. *282*, 20143085. https://doi.org/10.1098/rspb.2014.3085.

42. Scharschmidt, T.C., Vasquez, K.S., Truong, H.-A., Gearty, S.V., Pauli, M.L., Nosbaum, A., Gratz, I.K., Otto, M., Moon, J.J., Liese, J., et al. (2015). A Wave of Regulatory T Cells into Neonatal Skin Mediates Tolerance to Commensal Microbes. Immunity *43*, 1011–1021. https://doi.org/10.1016/j.immuni.2015.10.016.

43. Xie, N., Gong, H., Suhl, J.A., Chopra, P., Wang, T., and Warren, S.T. (2016). Reactivation of FMR1 by CRISPR/Cas9-Mediated Deletion of the Expanded CGG-Repeat of the Fragile X Chromosome. PLoS One *11*, e0165499. https://doi.org/10.1371/journal.pone.0165499.

44. Peter, C.J., Saito, A., Hasegawa, Y., Tanaka, Y., Nagpal, M., Perez, G., Alway, E., Espeso-Gil, S., Fayyad, T., Ratner, C., et al. (2019). In vivo epigenetic editing of Sema6a promoter reverses transcallosal dysconnectivity caused by C11orf46/Arl14ep risk gene. Nat. Commun. *10*, 4112. https://doi.org/10.1038/s41467-019-12013-y.

45. Colasante, G., Lignani, G., Brusco, S., Di Berardino, C., Carpenter, J., Giannelli, S., Valassina, N., Bido, S., Ricci, R., Castoldi, V., et al. (2020). dCas9-Based Scn1a Gene Activation Restores Inhibitory Interneuron Excitability and Attenuates Seizures in Dravet Syndrome Mice. Mol. Ther. *28*, 235–253. https://doi.org/10.1016/j.ymthe.2019.08.018.

46. Yamagata, T., Raveau, M., Kobayashi, K., Miyamoto, H., Tatsukawa, T., Ogiwara, I., Itohara, S., Hensch, T.K., and Yamakawa, K. (2020). CRISPR/dCas9-based Scn1a gene activation in inhibitory neurons ameliorates epileptic and behavioral phenotypes of Dravet syndrome model mice. Neurobiol. Dis. *141*, 104954. https://doi.org/10.1016/j.nbd.2020.104954.

47. Arsenault, J., Gholizadeh, S., Niibori, Y., Pacey, L.K., Halder, S.K., Koxhioni, E., Konno, A., Hirai, H., and Hampson, D.R. (2016). FMRP Expression Levels in Mouse Central Nervous System Neurons Determine Behavioral Phenotype. Hum. Gene Ther. *27*, 982–996. https://doi.org/10.1089/hum.2016.090.

48. Chen, C., Yuan, Y., O'Malley, H.A., Duba-Kiss, R., Chen, Y., Habig, K., Niibori, Y., Hodges, S.L., Hampson, D.R., and Isom, L.L. (2025). Neonatal but not juvenile gene therapy reduces seizures and prolongs lifespan in SCN1B–Dravet syndrome mice. J. Clin. Investig. *135*, e182584. https://doi.org/10.1172/JCI182584.

49. Vandesompele, J., De Preter, K., Pattyn, F., Poppe, B., Van Roy, N., De Paepe, A., and Speleman, F. (2002). Accurate normalization of real-time quantitative RT-PCR data by geometric averaging of multiple internal control genes. Genome Biol. 3, RESEARCH0034.

OMTM, Volume *33*

Supplemental information

Early postnatal expression mitigates immune responses to Cas9 in the murine central nervous system

Robert Duba-Kiss and David R. Hampson

Table S1. List of primers used in RT-qPCR gene expression experiments.

Target Gene	Forward Primer	Reverse Primer
Ppia	CGTCTCCTTCGAGCTGTTTG	CCACATGCTTGCCATCCAG
Pgk1	CTAGAGCTCCTGGAAGGTAAAG	GAGAGCTCAGCCTTTACAGC
S. aureus Cas9	GTACGGCGACGAGAAGAATC	ACTTGTACACGCCATTGTCC
Tnfα	CCCAAAGGGATGAGAAGTTCC	AGATAGCAAATCGGCTGACG
Il1β	TGCCACCTTTTGACAGTGATG	ATGTGCTGCTGCGAGATTTG
Ccl2	CCTGCTGCTACTCATTCACCA	CTTGAGCTTGGTGACAAAAACTACA
Ifnγ	ACTGGCAAAAGGATGGTGAC	GTGGGTTGTTGACCTCAAAC
Il10	GCTCTTACTGACTGGCATGAG	CGCAGCTCTAGGAGCATGTG
Tgfβ	TGGAAATCAACGGGATCAGC	TAGTTGGTATCCAGGGCTCTC

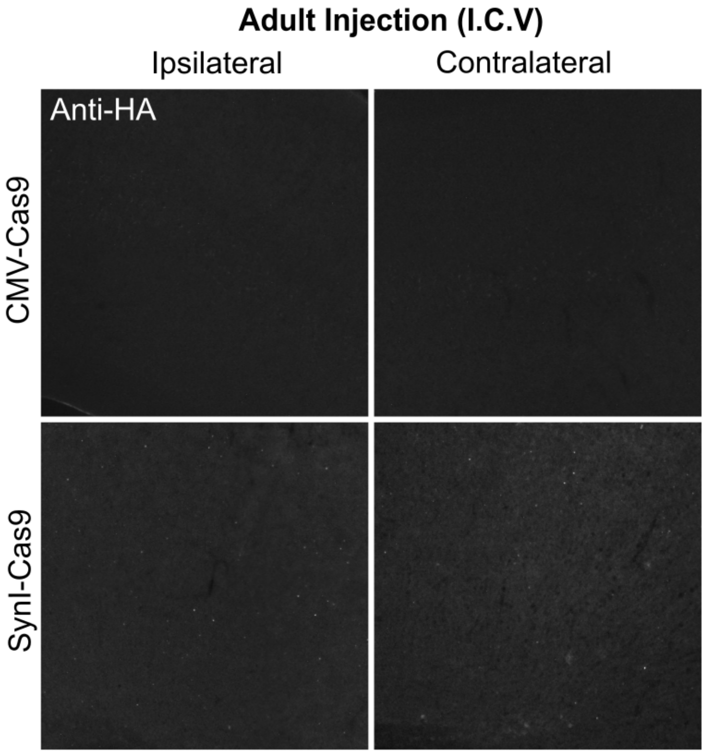

Figure S1. Representative low-magnification images showing lack of immunostaining using anti-HA in the somatosensory cortices of mice injected with Cas9-HA-encoding AAVs by i.c.v. injection as adults. Mice injected as neonates with AAV-SynI-Cas9 or AAV-CMV-Cas9 were used as positive controls for Cas9-HA expression analyses of adult-injected mice (not shown).

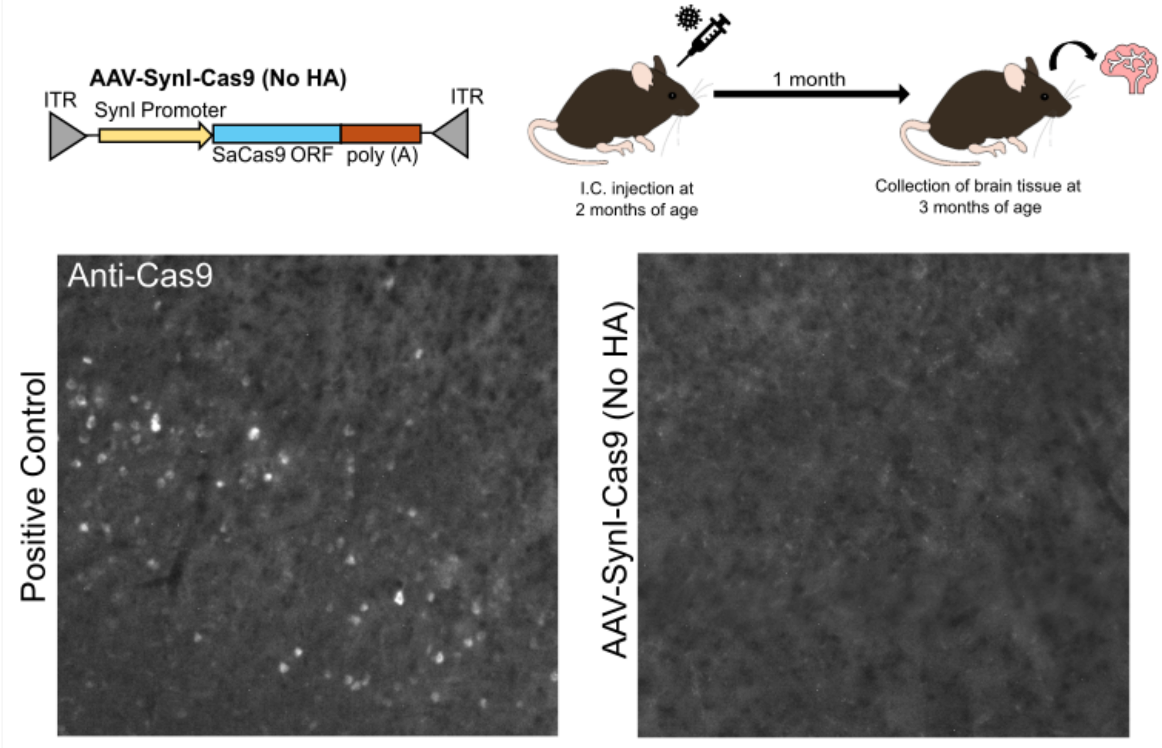

Figure S2. Representative low-magnification image of the somatosensory cortex of a mouse injected with AAV-SynI-Cas9 lacking an HA tag. Adult mice were given an intra-cortical injection with AAV-SynI-Cas9 (no HA) and the brains were collected 28 days post-injection (n = 6). Brain sections were probed with an anti-Cas9 antibody. The AAV dose used was 9.12 x 10^{10} GCs. The positive controls were brain sections from mice injected with AAV-SynI-Cas9 (with an HA tag) as neonates and were also probed with the anti-Cas9 antibody. HA: hemagglutinin; I.C.: intracortical; ITR: inverted terminal repeats; ORF: open reading frame.

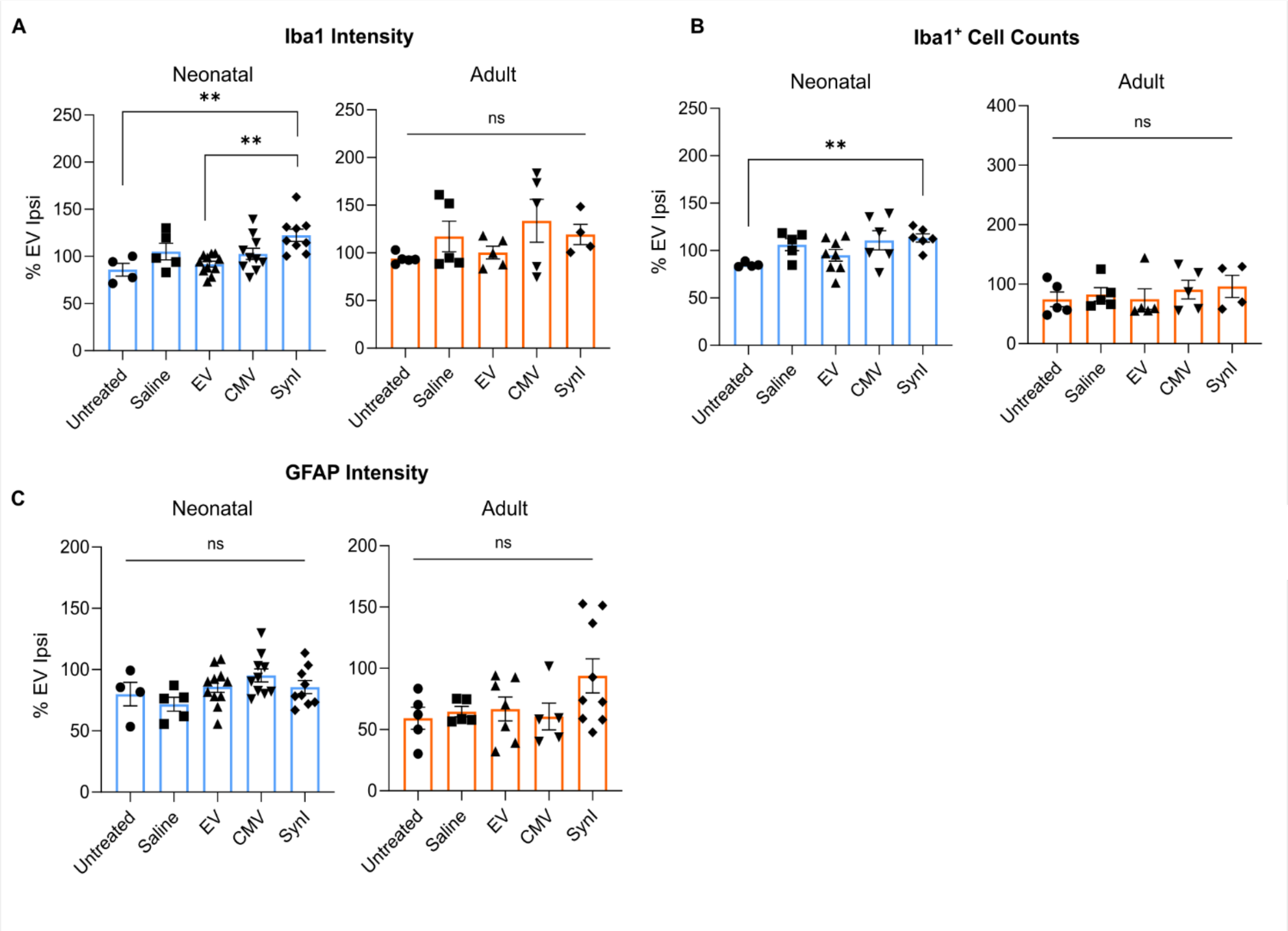

Figure S3. Quantitative analyses of Iba1 fluorescence intensity (A) and Iba1 cell counts (B) in the contralateral somatosensory cortices of mice administered AAV as neonates or adults. (C) Quantitative analyses of GFAP fluorescence intensity in the contralateral somatosensory cortices of mice administered AAV as neonates or adults. ns: not significant; ** p<0.01, Tukey *post hoc* or Dunnett's multiple comparison test.

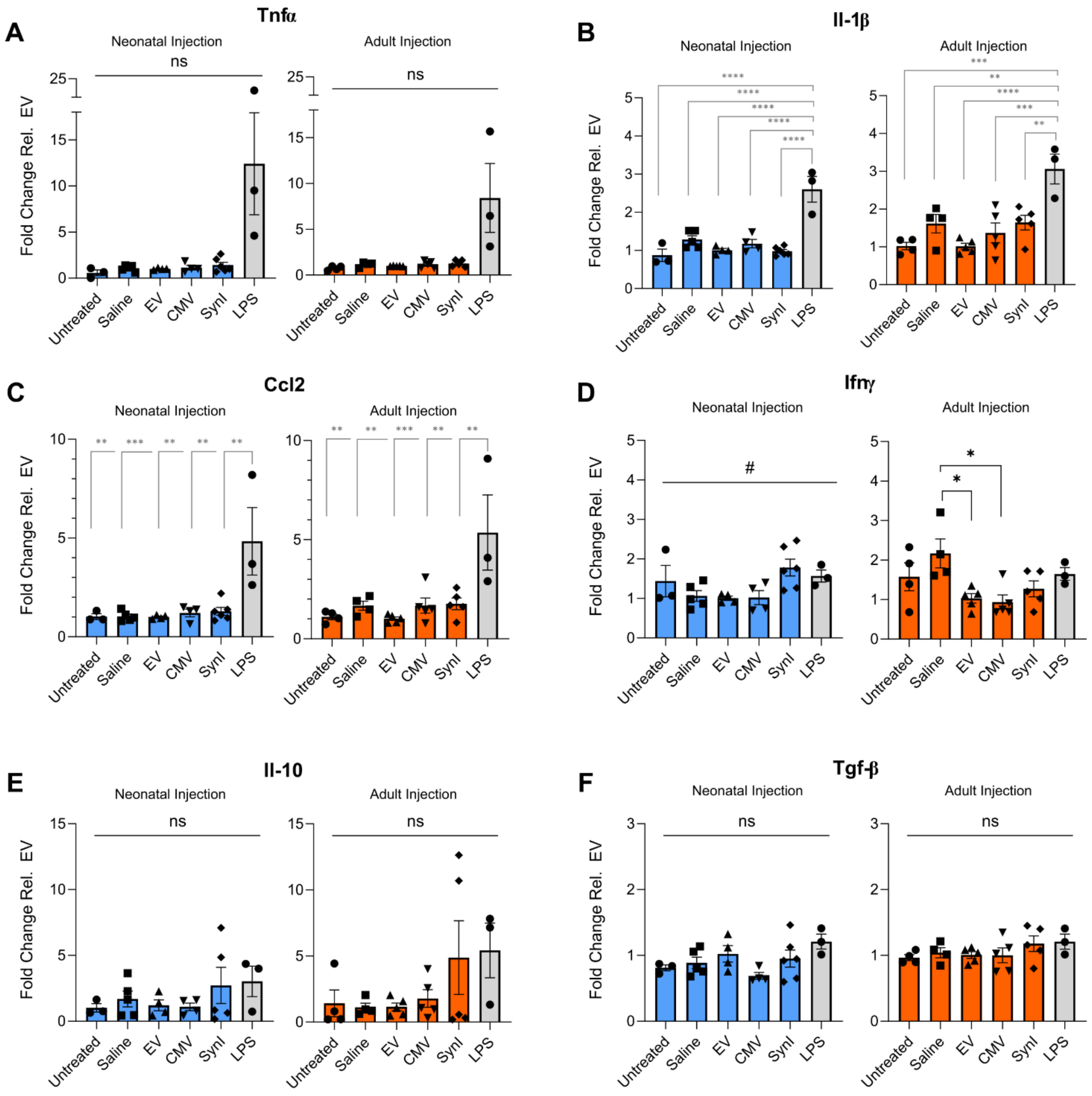

Figure S4. Quantification of cytokine expression in cerebral cortices one month following AAV treatment. Proinflammatory cytokines *Tnfα* (A), *Il-1β* (B), *Ccl2* (C), and *Ifnγ* (D), and anti-inflammatory cytokines *Tgf-β* (E) and *Il-10* (F). Cortical extracts from adult mice administered 2 mg/kg of LPS by intraperitoneal injection were used as a control (grey). ns: not significant; #: $p < 0.05$, one-way ANOVA; *: $p < 0.05$, **: $p < 0.01$, ***: $p < 0.001$, ****: $p < 0.0001$, Tukey's *post hoc* test.

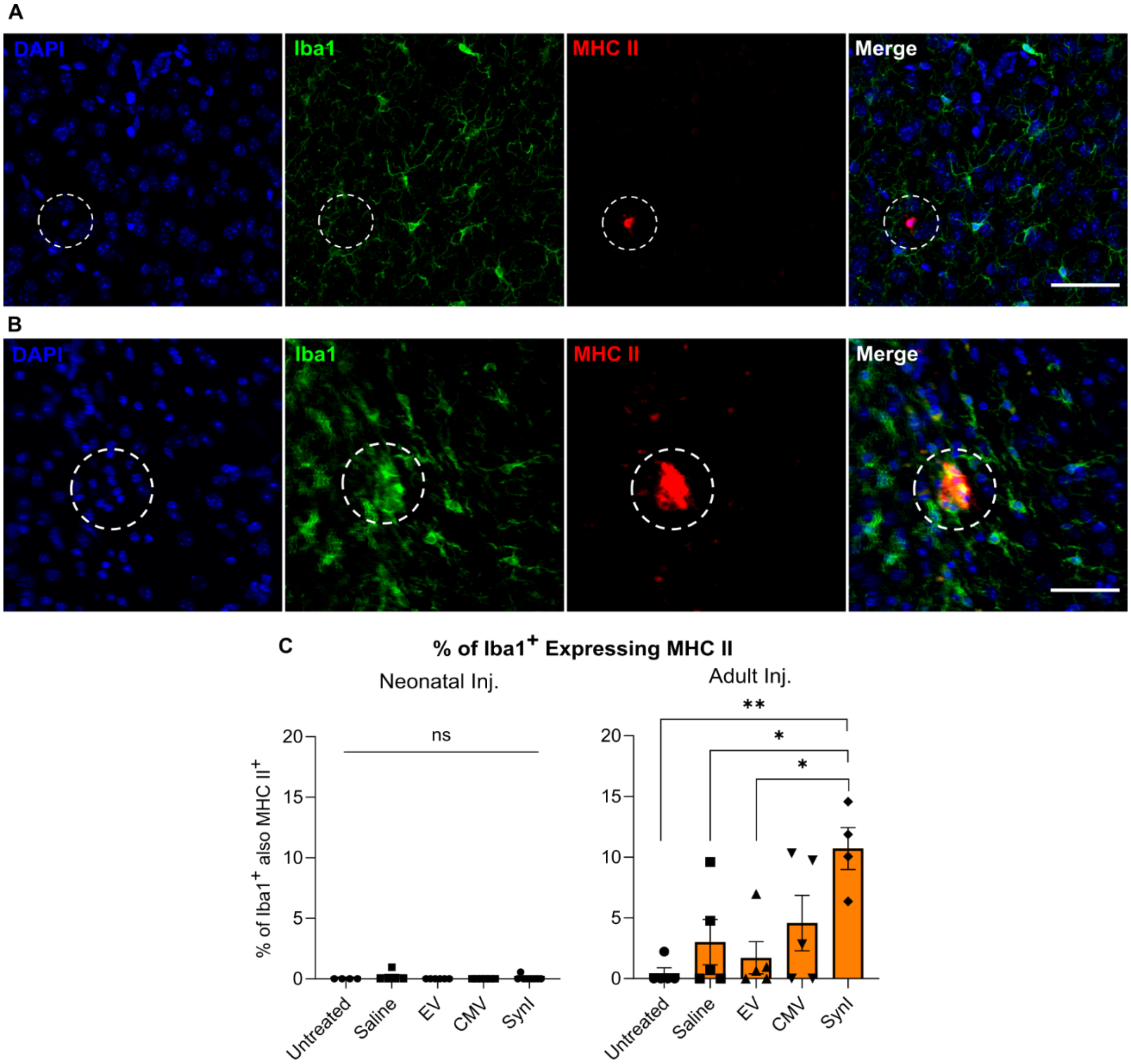

Figure S5. Representative images of double immunolabeling of Iba1 (green) and MHC II (red) in the ipsilateral somatosensory cortices of mice administered AAV-SynI-Cas9 as (A) neonates and (B) adults. Scale bars = 50 µm. (C) Quantitative results of co-labeling of $Iba1^+$ microglia and MHC II. ns: not significant; * $p < 0.05$, ** $p < 0.01$, Tukey's *post hoc* test.
